# Comparative transcriptomic analysis and functional characterization reveals that the class III peroxidase gene *TaPRX-2A* regulates drought stress tolerance in transgenic wheat

**DOI:** 10.3389/fpls.2023.1119162

**Published:** 2023-02-15

**Authors:** Peisen Su, Chao Sui, Yufei Niu, Jingyu Li, Shuhan Wang, Fanting Sun, Jun Yan, Shangjing Guo

**Affiliations:** ^1^ College of Agronomy, Liaocheng University, Liaocheng, China; ^2^ Key Laboratory of Huang-Huai-Hai Smart Agricultural Technology of the Ministry of Agriculture and Rural Affairs, College of Information Science and Engineering, Shandong Agricultural University, Tai’an, Shandong, China

**Keywords:** transcriptomics, drought tolerance, class III peroxidase, TaPRX-2A, ROS

## Abstract

Drought is a major abiotic stress that reduces crop yields and quality worldwide. Although some genes involved in the response to drought stress have been identified, a more in-depth understanding of the mechanisms underlying wheat tolerance to drought is needed for the control of drought tolerance. Here, we evaluated the drought tolerance of 15 wheat cultivars and measured their physiological–biochemical parameters. Our data showed that the drought tolerance of the resistant wheat cultivars was significantly higher than that of drought-sensitive cultivars, which was associated with a greater antioxidant capacity of the former. Transcriptomic analysis revealed that different mechanisms of drought tolerance exist between the wheat cultivars Ziyou 5 and Liangxing 66. Transcriptomic analysis also revealed a large number of DEGs, including those involved in flavonoid biosynthesis, phytohormone signalling, phenolamides and antioxidants. qRT–PCR was performed, and the results showed that the expression levels of *TaPRX-2A* were significantly different among the various wheat cultivars under drought stress. Further study revealed that overexpression of *TaPRX-2A* enhanced tolerance to drought stress through the maintenance of increased antioxidase activities and reductions in ROS contents. Overexpression of *TaPRX-2A* also increased the expression levels of stress-related genes and ABA-related genes. Taken together, our findings show that flavonoids, phytohormones, phenolamides and antioxidants are involved in the plant response to drought stress and that *TaPRX-2A* is a positive regulator of this response. Our study provides insights into tolerance mechanisms and highlights the potential of *TaPRX-2A* overexpression in enhancing drought tolerance in crop improvement programmes.

## Introduction

Environmental stresses severely impact plant growth, development and productivity. Drought is a major abiotic stress that reduces crop yields and quality worldwide and is occurring more frequently due to climate change and water scarcity (Mahajan and Tuteja, 2005; Zampieri et al., 2017). When persistently exposed to drought stress, plants can experience multiple types of damage (including oxidative injury) (Joshi et al., 2016). Reactive oxygen species (ROS), including superoxide radicals ( ${\text{O}}_{2}^{-}$ ), hydrogen peroxide (H_2_O_2_), and hydroxyl radicals (OH^−^), constitute the primary cause of oxidative damage, which damages cells by destroying membrane lipids (McCord, 2000). To counter environmental stresses, plants have evolved sophisticated molecular mechanisms, such as ROS scavenging, phytohormone signalling pathway (those of abscisic acid (ABA)) activation, and secondary metabolism (including that involving flavonoids, melatonin, and secoisolariciresinol) (Campo et al., 2014; Zhu, 2016; Meng et al., 2021; Feng et al., 2022; Song et al., 2022). Moreover, the antioxidant system plays important roles in reducing ROS levels by the action of several endogenous antioxidant enzymes (catalase (CAT), ascorbate peroxidase (APX), and superoxide dismutase (SOD)) (Mittler, 2002). In addition, by catalysing redox reactions, peroxidases (PRXs) protect cells from ROS damage (Wood et al., 2003).

PRXs are antioxidant enzymes that are widely distributed in living organisms, including plants, microorganisms, and animals. The members of the PRX superfamily are categorized into haem (animal and nonanimal) and nonheme PRXs based on their structure (Welinder, 1992; Taurog, 1999). In plants, PRXs can be divided into three classes (class I, class II, and class III) based on their functions. Class I PRXs include APXs, class II subgroups include lignin PRXs, and class III subgroups (guaiacol PRXs, EC1.11.17) include secretory PRXs that are plant specific (Bhatt and Tripathi, 2011). Currently, a large number of class III PRX families have been identified as multigenic families in plants. For example, there are 155 members of the class III PRX gene family in rice. *Arabidopsis thaliana* class III PRXs comprise 75 isoenzymes, and *Brachypodium distachyon* class III PRXs comprise 151 isoenzymes (Fawal et al., 2013). Maize class III PRXs comprise 119 members (Wang et al., 2015). In addition, 374, 159 and 169 PRX members have been identified in *Triticum aestivum*, *Triticum urartu* and *Aegilops tauschii*, respectively (Yan et al., 2019).

Class III subgroups comprise large multigene families in the plant kingdom, and these members are involved in various biophysiological processes, such as lignin biosynthesis, cell wall hardening, defence against pathogens, H2O2 removal, and tolerance to abiotic stresses (Cosio and Dunand, 2009; Wang et al., 2015). In plants, class III PRXs can oxidize phenolic compounds, thereby reducing the level of H2O2, which makes these enzymes efficient components of the antioxidant system against stresses. The function of members of the class III PRX gene family has been widely studied and demonstrated. In *A. thaliana*, *AtPrx71* is involved in cell growth, cell wall damage, and inhibition of cell expansion through accumulation of H_2_O_2_ (Raggi et al., 2015). At low temperature, the class III PRXs *PRX62* and *PRX69* promote root hair growth in *Arabidopsis* by modulating ROS homeostasis (Pacheco et al., 2022). It was reported that wheat PRX-1 is a specific wheat allergen that causes grass pollen-related wheat allergies (Ogino et al., 2021). Additionally, some class III PRXs respond to biotic and abiotic stresses. For example, *T. aestivum PRX111*, *PRX112* and *PRX113* respond to nematode infection (Simonetti et al., 2009). The French bean (*Phaseolus vulgaris*) PRX gene (*FBP1*) can cause oxidative burst impairments to increase *Arabidopsis* susceptibility to fungi and bacteria through the downregulation of *AtPrx33* and *AtPrx34* transcription (Daudi et al., 2012). In tomato, APX is phosphorylated by the protein kinase CPK28, which enhances thermotolerance (Hu et al., 2021). *OsPrx30* transcription is modulated by the AT-hook protein *OsATH1*, and its overexpression enhances rice susceptibility to rice bacterial blight through the maintenance of increased PRX activity and reductions of H_2_O_2_ contents (Liu et al., 2021). Overexpression of *AtPrx64* was shown to improve tolerance to aluminium stress through a reduction in ROS accumulation and increase in lignin content (Wu et al., 2017), and by mediating the IbBBX24-IbTOE3-IbPRX17 module, *IbPRX17* overexpression was shown to enhance abiotic stress tolerance (Zhang et al., 2022). Some studies have demonstrated that wheat glutathione peroxidase (GPXs) can improve salt tolerance in transgenic *Arabidopsis* (Zhai et al., 2013). Our previous study showed that, by scavenging ROS and regulating stress-related genes, *TaPRX-2A* can improve wheat salt tolerance (Su et al., 2020).

Wheat, an important commercial crop species, yields are often restricted by abiotic stresses. In this study, we evaluated the drought tolerance of various wheat cultivars and performed a transcriptomic analysis between Ziyou 5 (a drought-resistant line) and Liangxing 66 (a drought-sensitive line). Our results showed that drought stress altered several metabolic pathways, including those involving flavonoids, phytohormones, phenolamides and antioxidants. Furthermore, we functionally characterized the class III PRX gene *TaPRX-2A*. Overexpression of *TaPRX-2A* enhanced the tolerance to drought stress of the transgenic lines, which occurred through the scavenging of ROS and increase in stress-responsive gene transcription. This work provides researchers with new insights into the class III PRXs molecular mechanisms underlying drought stress tolerance of wheat.

## Materials and methods

### Plant materials and abiotic stress treatments

“Nonglin20”, “Shanrong 3”, “Jimai 22”, “Liangxing 66”, “Xiaoyan 6”, “Haomai 1”, “Ailiduo”, “Pubing 51”, “Qunzhong 10”, “Liangmai 2”, “LS117TL”, “Ziyou 5”, “Baokemai 1330”, “NSA98-1018” and “Pubing 9946” wheat (*T. aestivum*) seedlings were used in this study and obtained from Shandong Agricultural University. The seedlings were grown at 25°C under a photoperiod of 16/8 hours (h). The seedlings of the various wheat (*T. aestivum*) cultivars were subjected to drought stress when the plants produced one leaf and one heart. Concerning the drought stress treatment, the seedlings were not watered for 21 days (d) and then rewatered for 7 days.

### Transcriptional profiling

Ziyou 5 and Liangxing 66 wheat (*T. aestivum*) seedlings were treated with 20% PEG6000, and the leaves were harvested at 48 h after treatment. The RNA of the samples was extracted using an RNAprep Pure Plant Kit (TIANGEN) and sent to Metware Corporation (Wuhan) for RNA sequencing (RNA-seq), which was performed using an Illumina HiSeq™ 2000 instrument. The clean RNA-seq reads were subsequently mapped to the wheat reference genome (http://plants.ensembl.org/Triticum_aestivum/Info/Index). Then, the transcripts were assembled, and the differentially expressed genes (DEGs) were analyzed using the DEGseq R package with the default parameters (|log_2_(fold-change)| >= 1, false discovery rate (FDR) < 0.05) (Mortazavi et al., 2008).

### Isolation and cloning of *TaPRX-2A* and transgenic plant generation

We cloned the *TaPRX-2A* gene from the wheat cultivar Sumai 3. The wheat leaves were harvested from three-week-old plants and the RNA was extracted with TRIzol reagent (TransGen). The open reading frame (ORF) of *TaPRX-2A* (Ensembl ID: TraesCS2A02G573900) was obtained from the NCBI database (https://www.ncbi.nlm.nih.gov/). Specific primers were designed according to the *TaPRX-2A* sequence, and the primer sequences are listed in **Table S3**. Subsequently, the *TaPRX-2A* clone was ligated into the overexpression (OE) vector PC186 (pUbi::GWOE::Nos), after which the vector was transformed into the wheat KN199 background using particle gun-mediated gene transformation (Yao et al., 2006).

### Measurements of physiological–biochemical parameters

The leaves of various wheat cultivars were collected under control and drought stress treatment after 21 days. The all samples were used to measure physiological parameters related to drought stress, such as relative water content (RWC), malondialdehyde (MDA), ROS, and antioxidant enzymes. The following formula was used to measure the leaf RWC: RWC = (FW − DW)/(TW − DW) × 100% (FW, fresh weight; DW, dry weight; TW, turgid fresh weight) (Zhou et al., 2014). We used the thiobarbituric acid method to measure the MDA content (Heath and Packer, 1968), and we used the ninhydrin reaction method to measure the proline content and the anthrone method to measure the total soluble sugar content (Spiro, 1966; Bates et al., 1973). The ROS (H_2_O_2_, ${\text{O}}_{2}^{-}$ ) levels were visualized using 3,3’-diaminobenzidine (DAB) and nitroblue tetrazolium (NBT) staining (Gay et al., 1999; Tian et al., 2013). The activity of antioxidant enzymes (SOD, CAT, PRX) was measured using a previously described method (Chance and Maehly, 1955; Dhindsa et al., 1981; Aebi, 1984).

### Total RNA extraction and expression pattern analysis of stress-related genes

The RNA of leaves of various wheat cultivars, namely, “Shanrong 3”, “Liangxing 66”, “Xiaoyan 6”, “Ailiduo”, “Ziyou 5”, “Baokemai 1330”, “Shannong 41”, “JM22”, “Guomai 115”, “Taimai 198”, “Huaimai 33”, “NSA98-1018”, and “Pubing 9946”, *TaPRX-2A* transgenic lines and wild-type (WT) plants was extracted with TRIzol reagent (TransGen). Then, the mRNAs were reverse transcribed into first-strand cDNAs. The expression patterns of stress-related genes were determined using qRT–PCR (Roche LightCycler^®^ 480 system). The *18S rRNA* gene of wheat was used as an endogenous control, and the relative expression of the stress-related genes was calculated using the 2^–ΔΔCT^ method. All the qRT–PCR primers used in this study are listed in **Table S3**.

## Results

### Drought tolerance of different wheat cultivars

To comprehensively evaluate the drought tolerance of the different wheat cultivars, we assessed the drought tolerance of 15 different wheat cultivars. The seedlings of all the different wheat cultivars were subjected to drought stress for 21 days. Then, we measured their physiological–biochemical indices. The results showed that different wheat cultivars had different drought tolerances. Some wheat cultivars (Liangmai 2, LS117TL, Ziyou 5, Baokemai 1330, and NSA98-1018) were drought-tolerant and had high survival rates, while others (Nonglin 20, Shanrong 3, Jimai 22, Liangxing 66, Xiaoyan 6, Haomai 1 and Ailiduo) were drought-sensitive and had low survival rates. Similarly, several wheat cultivars (Qunzhong 10, Pubing 51, and Pubing 9946) were moderately drought tolerant. After drought stress for 21 days, the leaves of the drought-tolerant cultivars were slightly wilted compared with those of the drought-sensitive cultivars. The phenotypic changes and survival rates of different the wheat cultivars under both the control and the drought stress treatments were recorded (**Figure S1**).

**Figure 1:**
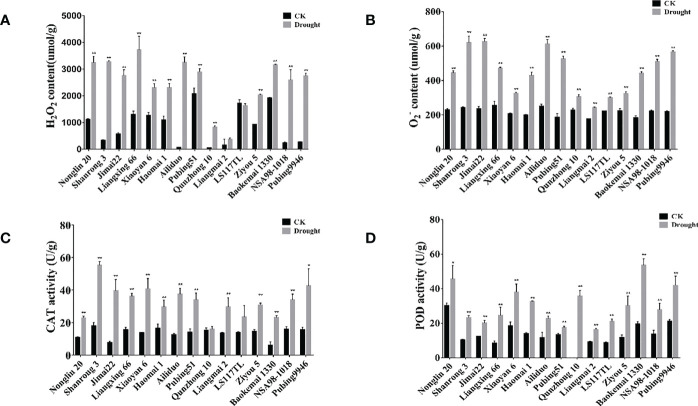
Analysis of ROS scavenging capacity and antioxidant enzymes activity. **(A)** H_2_O_2_ content for different wheat cultivars under control and drought treatment. **(B)** ${\text{O}}_{2}^{-}$ content for different wheat cultivars under control and drought treatment. **(C)** Detection of CAT activity for different wheat cultivars under control and drought treatment. **(D)** Detection of POD activity for different wheat cultivars under control and drought treatment. All experiments included three replicates and the data present the mean ± SD. *P < 0.05 and **P < 0.01 indicate a significant difference compared with WT.

ROS constitute the main cause of oxidative damage, which is associated with the physiological response to abiotic stresses in plants. To gain further insight into the differences in drought tolerance between different wheat cultivars, we measured several important physiological–biochemical properties associated with oxidation resistance. First, we measured the levels of ROS accumulation and the activities of antioxidant enzymes under drought stress. Under drought stress, we observed that the O^2-^ and H_2_O_2_ contents in the drought-tolerant wheat cultivars (Qunzhong 10, Liangmai 2, LS117TL, and Ziyou 5) were significantly lower than those in the drought-sensitive cultivars (Nonglin 20, Shanrong 3, Jimai 22, Liangxing 66, and Ailiduo) (**Figures 1A, B**). The activities of antioxidant enzymes (PRX, CAT) in the different wheat cultivars were subsequently measured. The results showed that the activities of PRX and CAT were higher in the drought-tolerant and drought-sensitive wheat cultivars under drought stress than in the controls. Specifically, the PRX activity in the drought-tolerant wheat cultivar Baokemai 1330 was significantly higher than that in the drought-sensitive cultivars (**Figures 1C, D**).

### Transcriptome sequencing analysis

To study the mechanism of drought tolerance between different wheat cultivars, we sequenced the transcriptome of a drought-tolerant wheat cultivar (Ziyou 5) and a drought-sensitive cultivar (Liangxing 66). The genes that were differentially between the control and treated samples were analysed. The results showed that 20663 (down, 8635; up, 12028) and 23775 (down, 12170; up, 11605) genes were differentially expressed in Liangxing 66 and Ziyou 5 (control vs. drought), respectively (**Figure S2A–C** and **Table S1**). According to our constructed Venn diagram, we focused on the 734 DEGs that cover 4 different combinations and found that these genes were mainly involved in the flavonoid, plant hormone, MAPK signalling, phenolamide and antioxidant pathways (**Figure S2C**; **Table S1**). Based on the DEG expression levels, principal component analysis (PCA) was performed on the samples (three biological replicates) (**Figure S2D**). According to the PCA score plot, the first principal components (PCs) accounted for 41.36% and the second PCs accounted for 18.47% of the total variance among the four sample groups. The four sample groups (i.e., Liangxing 66-CK, Liangxing 66-D, Ziyou 5-CK, Ziyou 5-D) could be easily distinguished from each other, since they were clustered into four distinct areas in the PCA score plot, indicating that each of the sample groups exhibited distinct expression profiles.

To determine the potential functions, all the DEGs were annotated by Gene Ontology (GO) enrichment analysis. The results showed that most DEGs were enriched in biological processes, including cellular processes (9319, 10610), metabolic processes (7824, 8884), responses to stimulus (5026, 5363), biological regulation (3623, 4040), and developmental processes (1930, 2099) in Liangxing 66 and Ziyou 5, respectively (control vs. drought). In the cellular component category, the top two categories were cellular anatomical entity (13490, 15364) and protein-containing complex (1239, 1603). In the molecular function category, the top four terms were binding (8968, 10076), catalytic activity (8639, 9664), transporter activity (1200, 1335), and transcription regulator activity (850, 917) (**Figures S3A, B**; **Table S1**).

To better understand the interactions of the DEGs, we performed a Kyoto Encyclopedia of Genes and Genomes (KEGG) (https://www.genome.jp/kegg) functional enrichment analysis of the DEGs (control vs. drought). The results showed that the related KEGG pathways were mostly enriched in metabolic pathways (3615, 4066), biosynthesis of secondary metabolites (2271, 2557), plant−pathogen interactions (962, 942), the MAPK signalling pathway (507, 517), plant hormone signal transduction (541, 570), starch and sucrose metabolism (346, 361), phenylpropanoid biosynthesis (319, 351), oxidative phosphorylation (164, 193), flavonoid biosynthesis (161, 179), glutathione metabolism (157, 189), peroxisomes (155, 182), tryptophan metabolism (106, 136), arginine and proline metabolism (92, 114), and flavone and flavonol biosynthesis (40, 55) in Liangxing 66 and Ziyou 5, respectively (control vs. drought) (**Figures S3C, D**; **Table S1**).

### Drought stress alters the expression profiles of genes involved in the flavonoid biosynthesis pathway

**Figure 2:**
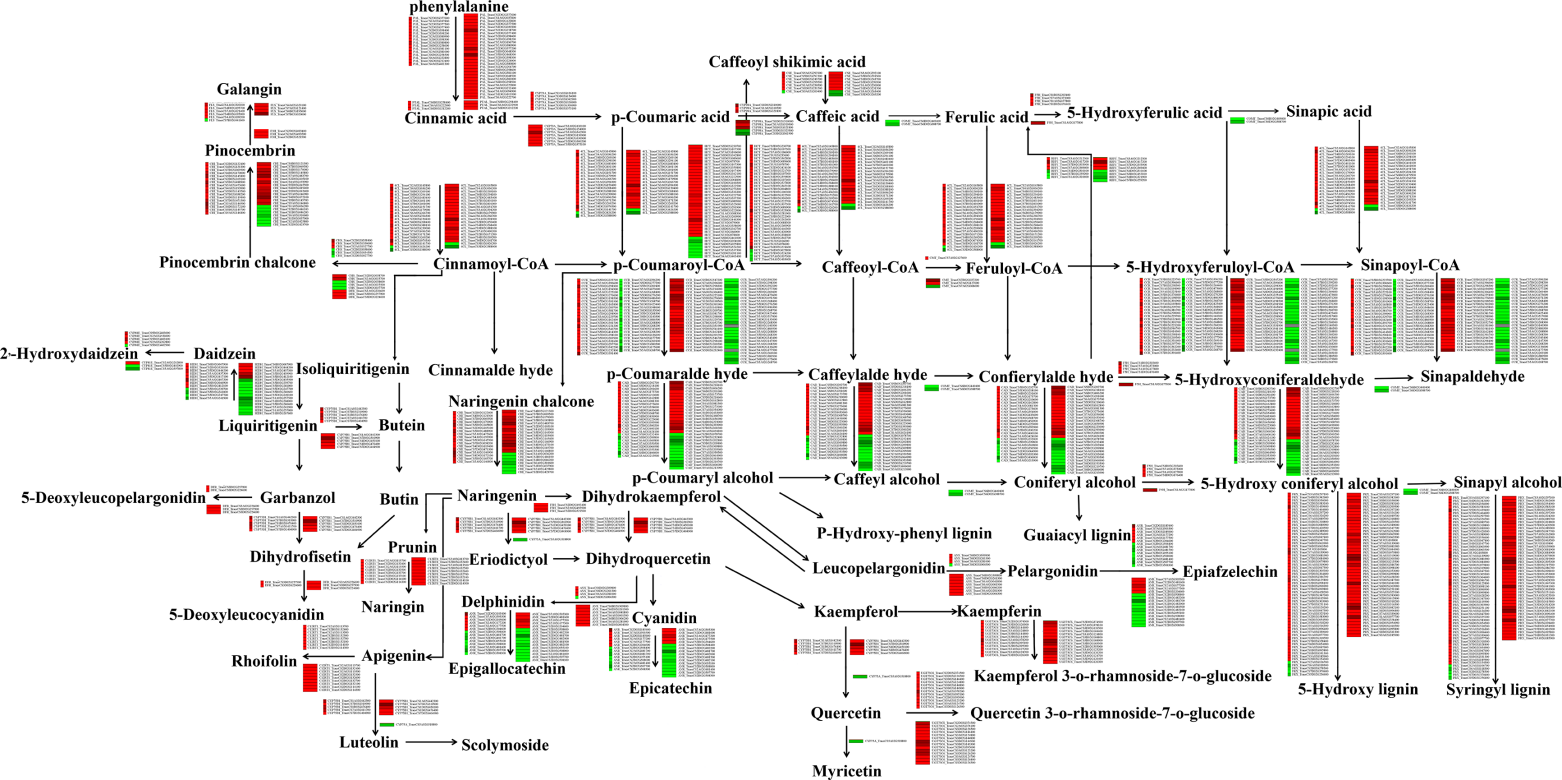
Effects of drought stress on flavonoid biosynthesis‐related genes. Regulatory network for the flavonoid metabolites, selected integrated expression levels of known genes involved in flavonoid biosynthetic pathway for wheat leaves treated with drought stress. The green and red represent doen-regulated and up-regulated gene expression under drought stress. *PAL*, phenylalanine ammonia-lyase; *PTAL*, phenylalanine/tyrosine ammonia-lyase; *CYP73A*, trans-cinnamate 4-monooxygenase; *COMT*, caffeic acid 3-O-methyltransferase; *CMT*, caffeoyl-CoA O-methyltransferase; *HCT*, shikimate O-hydroxycinnamoyltransferase; *CYP98A*, 5-O-(4-coumaroyl)-D-quinate 3’-monooxygenase; *CSE*, caffeoylshikimate esterase; *REF1*, coniferyl-aldehyde dehydrogenase; *DFR*, flavanone 4-reductase; *CYP75A*, flavonoid 3’,5’-hydroxylase; *PRX*, peroxidase; *4CL*, 4-coumarate–CoA ligase; *CCR*, cinnamoyl-CoA reductase; *HCT*, shikimate O-hydroxycinnamoyl transferase; *CYP98A*, 5-O-(4-coumaroyl)-D-quinate 3’-monooxygenase; *F5H*, ferulate-5-hydroxylase; *CAD*, cinnamyl-alcohol dehydrogenase; *CHS*, chalcone synthase; *CHI*, chalcone isomerase; *FLS*, flavonol synthase; *CYP75B1*, flavonoid 3’-monooxygenase; *ANS*, anthocyanidin synthase; *ANR*, anthocyanidin reductase; *HIDH*, 2-hydroxyisoflavanone dehydratase; *UGT73C6*, flavonol-3-O-L-rhamnoside-7-O-glucosyltransferase; *CYP81E*, 4’-methoxyisoflavone 2’-hydroxylase; *F3H*, naringenin 3-dioxygenase; *C12RT1*, flavanone 7-O-glucoside 2’’-O-beta-L-rhamnosyltransferase.

To better understand the influence of drought stress on the expression profiles of genes involved in the flavonoid biosynthesis pathway, we analysed the transcriptomic data of the drought-stressed wheat cultivars (Ziyou 5 and Liangxing 66). Based on the transcript levels, we constructed a network involving all the relationships of genes involved in the flavonoid biosynthesis pathway (**Figure 2**). The analysis showed that the expression levels of 372 (Liangxing 66-CK vs. Liangxing 66-D) and 405 (Ziyou 5-CK vs. Ziyou 5-D) genes in the flavonoid biosynthesis pathway changed. When combining data obtained from each group, we found that most expression levels of these genes increased between the two groups, as was the case for phenylalanine ammonia lyase (*PAL*), phenylalanine/tyrosine ammonia-lyase (*PTAL*), trans-cinnamate 4-monooxygenase (*CYP73A*), ferulate-5-hydroxylase (*F5H*), caffeoyl-CoA O-methyltransferase (*CMT*), 5-O-(4-coumaroyl)-D-quinate 3’-monooxygenase (*CYP98A*), flavanone 4-reductase (*DFR*), flavonoid 3’-monooxygenase (*CYP75B1*), and flavanone 7-O-glucoside 2’-O-beta-L-rhamnosyltransferase (*C12RT1*). However, the expression level of flavonoid 3’,5’-hydroxylase (*CYP75A*) decreased. In addition, the expression levels of 4-coumarate-CoA ligase (*4CL*), shikimate O-hydroxycinnamoyl transferase (*HCT*), cinnamoyl-CoA reductase (*CCR*), chalcone synthase (*CHS*), chalcone isomerase (*CHI*), anthocyanidin reductase (*ANR*), anthocyanidin synthase (*ANS*), *PRX*, and flavonol synthase (*FLS*) were different, with some increasing and some decreasing (**Table S2**). Considering that data are from the two different individual groups, the results of our transcriptomic analysis revealed specific increases in naringenin 3-dioxygenase (*F3H*) expression (Ziyou 5-CK vs. Ziyou 5-D) (**Figure 2**, **Table S2**). These results suggested that the flavonoid biosynthesis pathway in wheat plays a major role in the response to drought stress. Moreover, these results highlight that treatment of distinct wheat cultivars can consistently elicit discrete profiles of specific genes. In addition, the RNA-seq-based transcript levels of these genes involved in the flavonoid biosynthesis pathway were also verified *via* qRT‐PCR. The expression trends according to the qRT‐PCR data were consistent with the trends according to the transcriptomic data (**Figure S4**).

### Plant hormone signalling pathway activity is induced by drought stress

**Figure 3:**
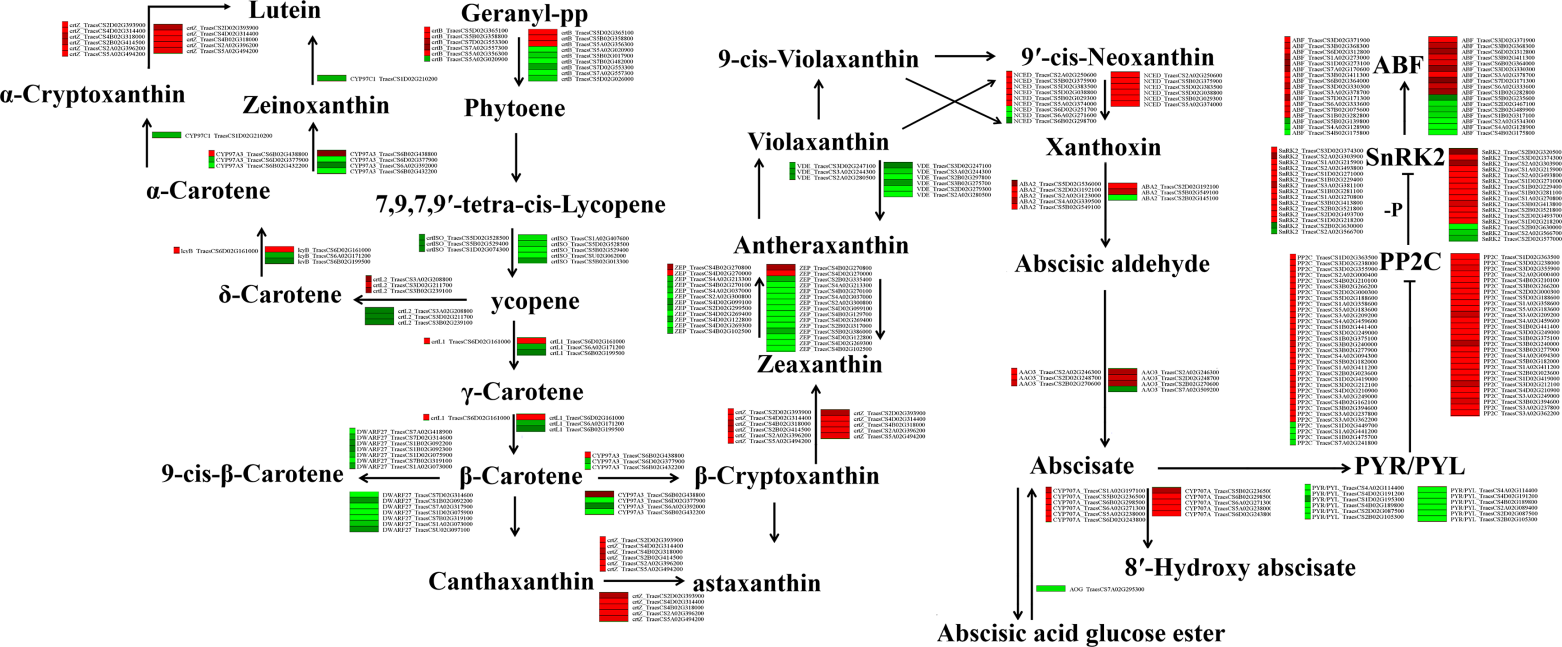
Effects of drought stress on ABA biosynthesis and signal transduction in wheat. Overview of ABA biosynthesis and signal transduction. The green and red represent down-regulated and up-regulated gene expression under drought stress. *ctrB*, 15-cis-phytoene synthase; *ctrZ*, beta-carotene 3-hydroxylase; *ZEP*, zeaxanthin epoxidase; *crtISO*, prolycopene isomerase; *crtL2*, lycopene epsilon-cyclase; *lcyB*, lycopene beta-cyclase; *CYP97A3*, beta-ring hydroxylase; *crtZ*, beta-carotene 3-hydroxylase; *CYP97C1*, carotenoid epsilon hydroxylase; *crtL1*, lycopene beta-cyclase; *DWARF27*, beta-carotene isomerase; *VDE*, violaxanthin de-epoxidase; *NCED*, 9-cis-epoxycarotenoid dioxygenase; *ABA2*, xanthoxin dehydrogenase; *AAO3*, abscisic-aldehyde oxidase; *AOG*, abscisate beta-glucosyltransferase; *PP2C*, protein phosphatase 2C; *CYP707A*, (+)-abscisic acid 8’-hydroxylase. *ABF*, ABA responsive element binding factor; *PYL*, abscisic acid receptor PYR/PYL family; *SnRK2*, serine/threonine-protein kinase SRK2.

Our transcriptome analysis also indicated that, in addition to the flavonoid biosynthesis pathway, the ABA (a plant hormone) pathway is involved in the response to drought stress. Therefore, we generated a network to explore the relationships between drought stress and ABA signalling pathways. Under drought stress, 148 (Liangxing 66-CK vs. Liangxing 66-D) and 166 (Ziyou 5-CK vs. Ziyou 5-D) ABA-related genes were enriched in plant hormone pathways. The expression levels of beta-carotene 3-hydroxylase (*crtZ*), xanthoxin dehydrogenase (*ABA2*), and abscisic-aldehyde oxidase (*AAO3*) increased, while those of prolycopene isomerase (*crtISO*), violaxanthin de-epoxidase (*VDE*), and PYR/PYL decreased. Moreover, we found that carotenoid epsilon hydroxylase (*CYP97C1*) was specifically expressed in the samples (Ziyou 5-CK vs. Ziyou 5-D) (**Figure 3**; **Table S2**). Similarly, the expression trends of genes involved in the ABA pathway according to the qRT‐PCR data were consistent with the trends according to the transcriptomic data (**Figure S5**).

We also generated networks about auxin, SA and JA pathways. (1) auxin: Under drought stress, 171 (Liangxing 66-CK vs Liangxing 66-D) and 210 (Ziyou 5-CK vs Ziyou 5-D) auxin-related genes were enriched in plant hormone pathways. Among them, the expression levels of 3-phosphoshikimate 1-carboxyvinyltransferase (*AROA*), chorismate synthase (*AROC*), anthranilate phosphoribosyl transferase (*TRPD*), aromatic aminotransferase (*ISS1*), and transport inhibitor response 1 (*TIR1*), exhibited upregulation, while arylalkylamine N-acetyltransferase (*AANAT*), indole-3-acetaldehyde oxidase (*AAO*) exhibited downregulation. Indole-3-pyruvate monooxygenase (*YUCCA*), amidase (*AMI*), L-tryptophan—pyruvate aminotransferase (*TAA1*), auxin-responsive protein (*AUX/IAA*), auxin response factor (*ARF*), auxin responsive GH3 gene family (*GH3*), SAUR family protein (*SAUR*) were different expression trends (upregulations, or downregulations). (2) SA: Under drought stress, 46 (Liangxing 66-CK vs Liangxing 66-D) and 59 (Ziyou 5-CK vs Ziyou 5-D) genes in SA pathways were enriched in plant hormone pathways. Among them, the expression levels of *PAL*, *PTAL*, and *PR1* were significantly upregulated, whereas *ICS* and *NPR1* were significantly downregulated. (3) JA: Under drought stress, 179 (Liangxing 66-CK vs Liangxing 66-D) and 168 (Ziyou 5-CK vs Ziyou 5-D) genes in JA pathway were enriched in plant hormone pathways. Among them, the expression levels of hydroperoxide dehydratase (*AOS*), allene oxide cyclase (*AOC*), and acetyl-CoA acyltransferase (*FAD*) were upregulated, whereas hydroperoxide lyase (*HPL*) was significantly downregulated. Fatty acid alpha-dioxygenase (*DOX*) was significantly upregulated in “Liangxing 66” (control vs. drought treated), while *DOX* was significantly downregulated in “Ziyou 5” (control vs. drought treated). OPC-8:0 CoA ligase 1 (*OPCL1*) and jasmonate O-methyltransferase (*JAM*) specially expressed in “Liangxing 66” (control vs. drought treated), while *COI1* specially expressed in “Ziyou 5” (control vs. drought treated) (**Figure S6**, **Table S2**). In addition, qRT-PCR analysis confirmed the significantly differential gene expression trends detected in transcriptome data (**Figure S7**).

### Phenolamides and antioxidant pathway activity in response to drought stress

**Figure 4:**
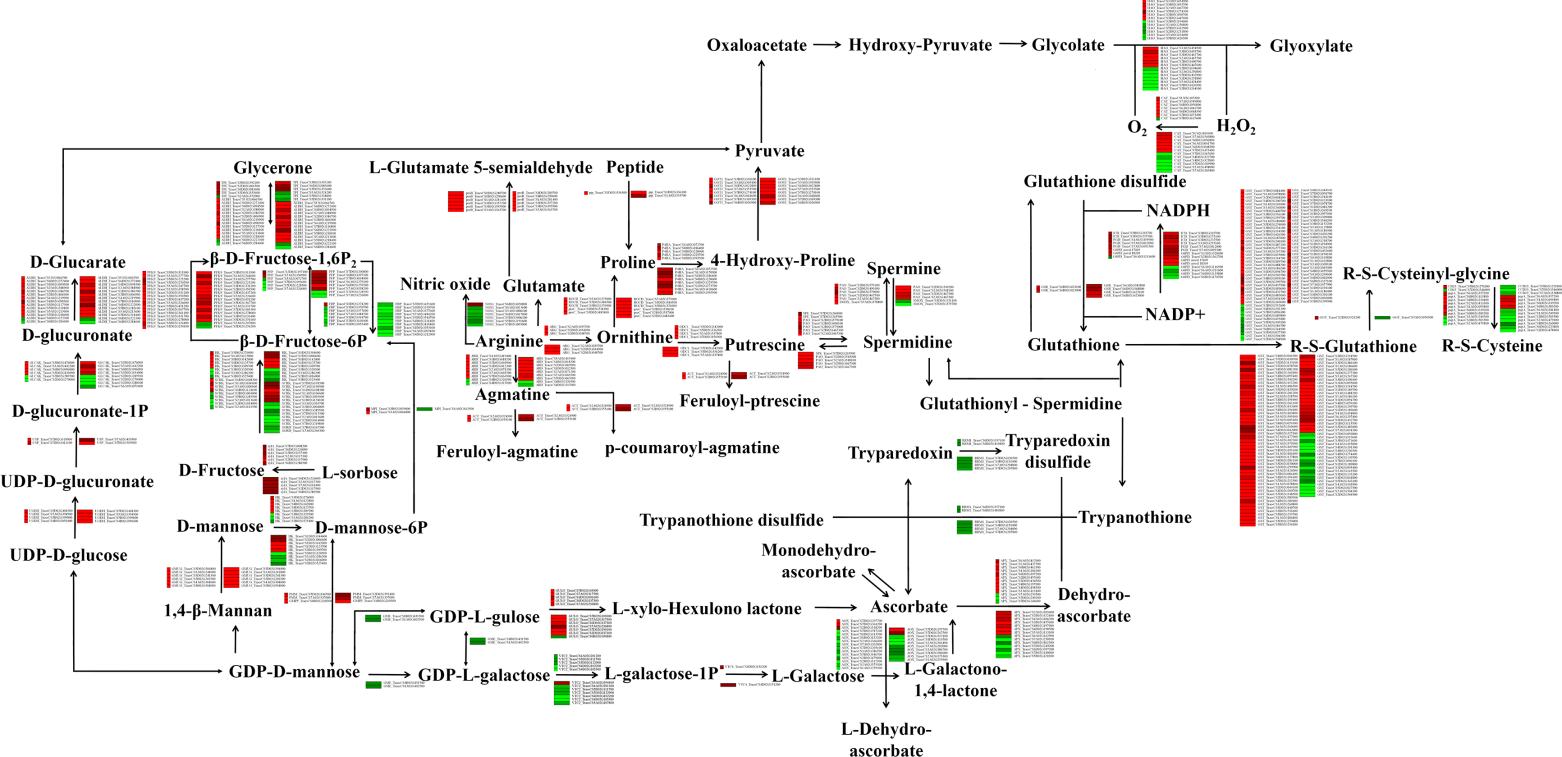
Effects of drought stress on phenolamides and antioxidant biosynthesis-related genes in wheat. Overview of phenolamides and antioxidant biosynthesis. The green and red represent doen-regulated and up-regulated gene expression under drought stress. *ALDH*, aldehyde dehydrogenase (NAD+); *SMOX*, spermine oxidase; *ROCD*, ornithine–oxo-acid transaminase; *ACT*, agmatine coumaroyltransferase; *PRDX6*, peroxiredoxin 6; *G6PD*, glucose-6-phosphate 1-dehydrogenase; *GPX*, glutathione peroxidase; *APX*, L-ascorbate peroxidase; *RRM1*, ribonucleoside-diphosphate reductase subunit M1; *NOS1*, nitric-oxide synthase; *proB*, glutamate 5-kinase; *proC*, pyrroline-5-carboxylate reductase; *pip*, proline iminopeptidase; *P4HA*, prolyl 4-hydroxylase; *GOT1*, aspartate aminotransferas; *ODC1*, ornithine decarboxylase; *GLDH*, L-galactono-1,4-lactone dehydrogenase; *SPE*, spermidine synthase; *GSR*, glutathione reductase (NADPH); *PGD*, 6-phosphogluconate dehydrogenase; *ICD*, isocitrate dehydrogenase; *GGT*, gamma-glutamyltranspeptidase/glutathione hydrolase; *UGDH*, UDP glucose 6-dehydrogenase; *GMPP*, mannose-1-phosphate guanylyltransferase; *SORD*, L-iditol 2-dehydrogenase; *xylA*, xylose isomerase; *RRM1*, ribonucleoside-diphosphate reductase subunit M1; *GST*, glutathione S-transferase; *CD13*, aminopeptidase N; *pepA*, leucyl aminopeptidase; *GME*, GDP-D-mannose 3’, 5’-epimerase; *VTC2*, GDP-L-galactose phosphorylase; *VTC4*, inositol-phosphate phosphatase; *GULO*, L-gulonolactone oxidase; *AOX*, L-ascorbate oxidase; *NADH*, monodehydroascorbate reductase; *HK*, hexokinase; *scrK*, fructokinase; *PFP*, diphosphate-dependent phosphofructokinase; *PFK9*, 6-phosphofructokinase; *FBP*, fructose-1,6-bisphosphatase I; *ALDO*, fructose-bisphosphate aldolase; *TPI*, triosephosphate isomerase; *PMM*, phosphomannomutase; *GMUG*, mannan endo-1,4-beta-mannosidase; *MPI*, mannose-6-phosphate isomerase; *USP*, UDP-sugar pyrophosphorylase; *GLCAK*, glucuronokinase; *HAO*, (S)-2-hydroxy-acid oxidase; *CAT*, catalase.

We found that drought stress modulates the phenolamide and antioxidant pathways. The transcriptomic data revealed significant differences in the expression of many phenolamide-related and antioxidant pathway-related genes. Among them, arginase (*ARG*), agmatine coumaroyl transferase (*ACT*), glutamate 5-kinase (*proB*), pyrroline-5-carboxylate reductase (*proC*), ornithine–oxo-acid transaminase (*ROCD*), prolyl 4-hydroxylase (*P4HA*), aspartate aminotransferase (*GOT1*), ornithine decarboxylase (*ODC1*), spermidine synthase (*SPE*), polyamine oxidase (*PAO*), phosphomannomutase (*PMM*), and mannose-1-phosphate guanylyltransferase (*GMPP*) were upregulated in response to drought stress. In contrast, ribonucleoside-diphosphate reductase subunit M1 (*RRM1*) was significantly downregulated under drought stress. Moreover, the expression trends of genes involved in the antioxidant pathway, such as *GPX*, L-*APX*, peroxiredoxin (*PRDX6*), glutathione reductase (*GSR*), gamma-glutamyltranspeptidase (*GGT*), glutathione S-transferase (*GST*), L-ascorbate oxidase (*AOX*), monodehydroascorbate reductase (*NADH*), (S)-2-hydroxy-acid oxidase (*HAO*), and *CAT*, were different, with the expression of some increasing and some decreasing, in the Liangxing 66-CK vs. Liangxing 66-D and Ziyou 5-CK vs. Ziyou 5-D comparison groups. GDP-D-mannose 3’,5’-epimerase (*GME*) was expressed specifically in Ziyou 5 (control vs. drought stressed) (**Figure 4**; **Table S2**). We also compared the expression levels of the genes involved in the phenolamide and the antioxidant pathways between the qRT‐PCR data and the transcriptomic data, and the we found that the expression trends were also the same (**Figure S8**).

### Coexpression analysis

**Figure 5:**
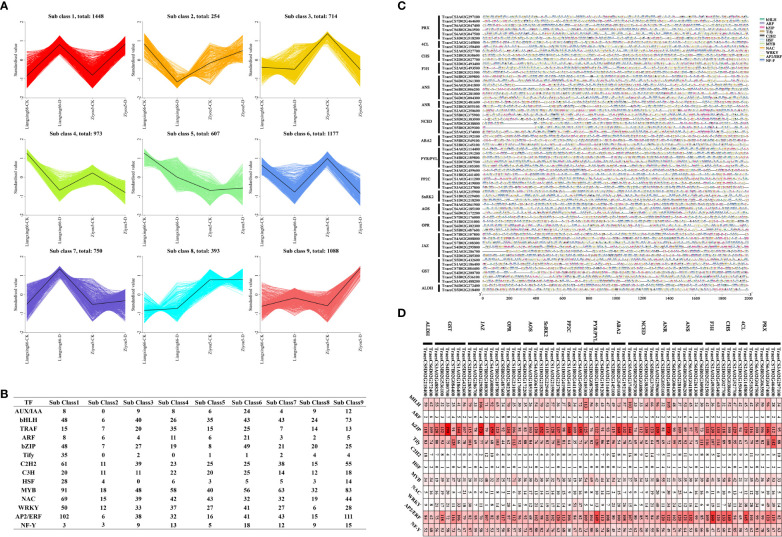
**(A)** Cluster analysis of co-expression patterns of genes associated with flavonoid, phytohormone, phenolamides and antioxidant metabolic pathways and all differentially expressed transcription factors. **(B)** Quantity statistics of the transcription factors in different sub clusters. **(C)** The promoter binding sites analysis of genes associated with different pathways. **(D)** The cis-elements distribution. We analyzed transcription factor binding site based on the 2000 bp DNA sequence upstream of the gene.

To explore the regulatory mechanisms of genes involved in different metabolic pathways, we performed a coexpression cluster analysis of these genes involved in different metabolic pathways and all differentially expressed transcription factor (TF)-encoding genes. We found that these DEGs could be divided into 9 clusters: subclass 1-subclass 9 (**Figure 5A**). We performed an additional analysis to explore which types of TFs have regulatory functions with respect to these genes among various metabolic pathways and counted the number of TF-encoding genes in the 9 clusters. The results showed that bHLH, bZIP, NAC, C2H2, MYB and AP2/ERF TFs were the most abundant (**Figure 5B**). We analysed the TF-binding sites of these gene promoters in the 2000 bp upstream sequences, and the results showed that the binding sites of bHLH, bZIP, Tify, MYB, ERF, and NF-Y TFs throughout the promoter regions of these genes were the most abundant, including in the *ALDH*, *GST*, *JAZ*, *OPR*, *AOS*, *SnRK2*, *PP2C*, *PYL*, *ABA2*, *NCED*, *ANR*, *ANS*, *F3H*, *CHS*, *4CL* and *PRX* genes (**Figures 5C, D**). Taken together, these results suggested that the regulation of TFs and pathway genes affects the response to drought stress in wheat.

### *TaPRX-2A* enhances the drought tolerance of transgenic wheat

**Figure 6:**
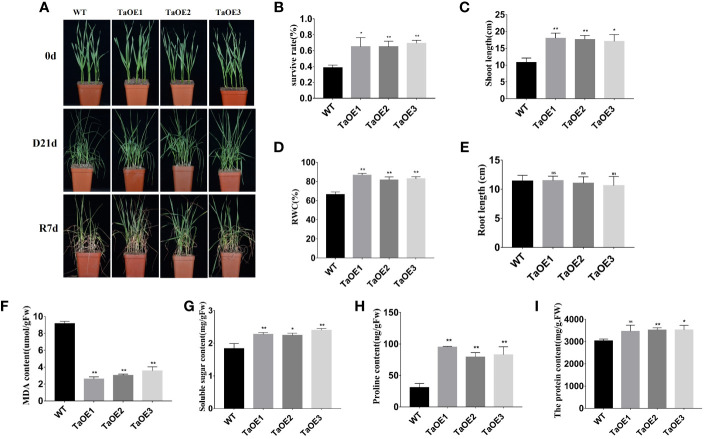
*TaPRX-2A* overexpression increased the drought tolerance. **(A)** Phenotype of *TaPRX-2A*-overexpressing transgenic and WT wheat (the cultivar “KN199”) with drought treatment. **(B)** Survival rates of *TaPRX-2A*-overexpressing transgenic lines and WT wheat. **(C)** shoot length of *TaPRX-2A*-overexpressing transgenic lines and WT wheat. **(D)** Relative water content (RWC), and **(E)** root length. **(F)** MDA content of *TaPRX-2A*-overexpressing transgenic lines and WT wheat. **(G)** soluble sugar content of *TaPRX-2A*-overexpressing transgenic lines and WT wheat. **(H)** proline content, and **(I)** soluble protein content of *TaPRX-2A*-overexpressing and WT plants. All experiments included three replicates and the data present the mean ± SD. *P < 0.05 and **P < 0.01 indicate a significant difference compared with WT. The “ns” presents “no differences”.

PRXs have been shown to be involved in plant tolerance to abiotic stresses. Our RNA‐seq data indicated that the expression levels of many PRXs changed after drought stress treatment. In our previous study, we confirmed that a PRX gene, *TaPRX-2A*, responded to various abiotic stresses (salt and drought), and *TaPRX-2A* overexpression was shown to enhance the salt tolerance of wheat significantly (Su et al., 2020). However, what’s interesting is that this gene *TaPRX-2A* is not found in our transcriptome. To further explore the detailed temporal and spatial expression patterns of *TaPRX-2A* at different time after drought treatment, we measured the expression levels of *TaPRX-2A* in the wheat cultivars with varying degrees of drought tolerance at 24h and 48h after drought treatment. Compared with that of “Liangxing 66”, the expression profile of *TaPRX-2A* was significantly different in several wheat cultivars with different drought tolerances, such as “Ziyou 5”, “Xiaoyan 6”, “Guomai 115”, and “Baokemai 1330”, at 24 h after treatment (**Figure S9**). Thus, we grew three independent *TaPRX-2A* overexpression transgenic lines (TaOE1, TaOE2, and TaOE3) and WT wheat plants under natural drought-stress conditions for 21 days, followed by rewatering for 7 days. Under nonstress conditions, we observed no significant differences in phenotypic characteristics between the *TaPRX-2A* transgenic lines and WT wheat plants. However, the *TaPRX-2A* transgenic lines grew more robustly than did the WT plants. The WT plants were more withered after drought stress treatment for 21 days. After being rewatered for 7 days, the *TaPRX-2A* transgenic lines presented higher survival percentages (65%, 65.2, 69.3%) than did the WT plants (38.7%) (**Figures 6A, B**). Then, the shoot length, RWC, and root length of the TaOE and WT plants were compared between the normal and drought-stress conditions. Compared with the WT plants, the TaOE plants had longer shoots and a higher RWC (**Figures 6C, D**). Interestingly, the lengths of the roots of the TaOE plants were not significantly different from those of the WT plants (**Figure 6E**). Taken together, these results indicated that *TaPRX-2A* overexpression drastically enhanced wheat drought tolerance.

### *TaPRX-2A* overexpression influences physiological-biochemical indices related to osmotic and oxidative stress

We further measured physiological–biochemical indices, including MDA, soluble sugar, proline, and soluble protein contents, to explore the mechanism of *TaPRX-2A*-mediated drought resistance. The results showed that the MDA, soluble sugar, proline, and soluble protein contents in the WT were not significantly different from those of the TaOE lines under normal conditions. However, the MDA content in the WT was significantly higher than that in the TaOE lines under drought stress (**Figure 6F**). The soluble sugar, proline, and soluble protein contents in the WT were significantly lower than those in the TaOE lines (**Figures 6G–I**). These results demonstrated that *TaPRX-2A* overexpression improved transgenic plant tolerance to drought stress through changes to metabolite (MDA, soluble sugar, proline, and soluble protein) contents (**Figure 6**).

### *TaPRX-2A* regulates ROS scavenging in transgenic wheat

**Figure 7:**
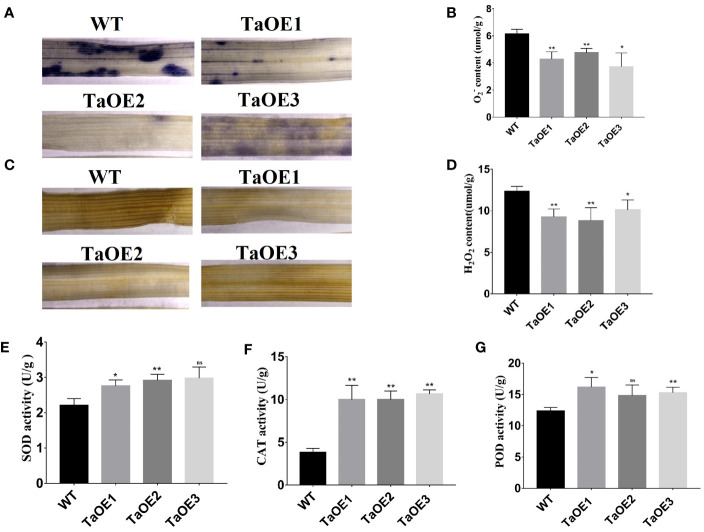
Analysis of ROS scavenging capacity andantioxidant enzymes activity in transgenic wheat lines. **(A)** Detection of ${\text{O}}_{2}^{-}$ generation by NBT staining and ${\text{O}}_{2}^{-}$ content **(B)**. **(C)** Detection of H_2_O_2_ accumulation by DAB staining and H_2_O_2_ content **(D)**. **(E)** Detection of SOD activity in *TaPRX-2A*-overexpressing transgenic lines and WT wheat. **(F)** Detection of CAT activity in *TaPRX-2A*-overexpressing transgenic lines and WT wheat. **(G)** Detection of POD activity in *TaPRX-2A*-overexpressing transgenic lines and WT wheat. All experiments included three replicates and the data present the mean ± SD. *P < 0.05 and **P < 0.01 indicate a significant difference compared with WT. The “ns” presents “no differences”.

PRXs can scavenge ROS to maintain steady-state levels of ROS when plants experience stress conditions. Therefore, the ROS levels of the TaOE and WT lines under drought stress were assessed. The levels of ${\text{O}}_{2}^{-}$ and H_2_O_2_ were major indicators of the ROS level. Therefore, the accumulation of ${\text{O}}_{2}^{-}$ and H_2_O_2_ was measured in both the TaOE and the WT lines. The accumulation of ${\text{O}}_{2}^{-}$ and H_2_O_2_ was determined by NBT staining and DAB staining. The results showed that the levels of ${\text{O}}_{2}^{-}$ (stained blue with NBT) and H_2_O_2_ (stained brown by DAB) were significantly higher in the WT plants than in the transgenic lines under drought stress (**Figures 7A–D**). In addition, we measured the activities of antioxidant enzymes (SOD, PRX, and CAT). The results showed that the SOD, CAT, and PRX activities in the transgenic plants were significantly higher than those in the WT plants (**Figures 7E–G**).

### *TaPRX-2A* regulates the expression of stress-related genes

To determine whether *TaPRX-2A* affects the expression of stress-related genes that contribute to drought tolerance, the expression patterns of various stress-related genes were determined in the transgenic lines and WT plants (**Figure S10**). These stress-related genes were selected on the basis of their purported involvement in the response to various abiotic stresses. These genes include *TLP4*, which encodes a thaumatin-like protein; *DE6*, which encodes dehydrin 6; *RD22*, which encodes a dehydration-responsive protein; ABA-related genes including *CRTB*, *ZEP*, *PYR*, *PP2c*, and *SnRK2*; *ABAI*, which encodes an ABA-inducing protein; *GLP4*, which encodes a germin-like protein; *GST22*, which encodes glutathione S-transferase; and *FeSOD*, *CuSOD*, *APX*, and *CAT*, which encode ROS-scavenging enzymes. We found that several of these genes (*RD22*, *ABAI, CAT* and *APX*) were highly expressed in the transgenic lines and in the WT plants under both nonstress and drought-stress conditions. In addition, the expression of several stress-related genes (*TLP4*, *GLP4*, *GST22*, *FeSOD*, *CuSOD*) was higher in the transgenic lines than in the WT plants under drought stress. Nevertheless, the expression of the *DE6* gene was not significantly different between the transgenic lines and the WT plants (**Figure S10A**). In addition, we identified ABA-related genes, including *CRTB*, *ZEP*, *PYR*, *PP2c*, and *SnRK2*, in the transgenic lines and WT plants, and the results showed that the expression levels of these ABA-related genes were higher in the transgenic lines than in the WT plants under drought stress. Other genes (*ZEP*, *PYR*) were expressed as low levels in both the transgenic lines and in the WT plants under drought stress (**Figure S10B**). Taken together, these results suggested that the overexpression of *TaPRX-2A* improved drought tolerance by the control of stress-responsive and ABA-related gene expression.

## Discussion

Drought stress is a widespread abiotic stress that causes major crop yield and economic losses (Mahajan and Tuteja, 2005; Zampieri et al., 2017). The molecular mechanisms through which plants respond to drought conditions have been extensively reported. Plants increase their tolerance to drought stress by activating signalling pathways that drive biochemical and physiological responses. Secondary metabolism, including the production of flavonoids, melatonin, secoisolariciresinol and plant hormones (ABA), is a major signalling event (Campo et al., 2014; Zhu, 2016; Meng et al., 2021; Feng et al., 2022; Song et al., 2022). Class III PRXs compose a large gene family with multiple genes in higher plants. It has been reported that class III PRXs respond to abiotic stresses. However, information regarding class III PRX resistance mechanisms used by wheat to combat drought stress is limited. Here, we revealed that, by scavenging ROS and enhancing stress-responsive gene expression, *TaPRX-2A* enhanced wheat tolerance to drought conditions.

Many studies have revealed that integrated analysis of transcriptomic data can yield functional insights into various biological processes (Hoefgen and Nikiforova, 2008). It is also known that flavonoid biosynthesis, plant hormonal signalling, and peroxidases play an important role in plant against abiotic stresses (Ma et al., 2014; Guo et al., 2020). Like these studies, our study revealed changes in flavonoids, plant hormones, phenolamides and activity of antioxidant signalling pathways, thereby expanding the scope of known drought tolerance‐related DEGs. The previous study showed that PRXs enhanced wheat tolerance against salt stress through ABA signalling (Su et al., 2020). As components with antioxidant properties, the flavonoids and peroxidases has been widely reported to be involved in plant resistance to abiotic stress, respectively. But, little information has been showed about the regulatory relationship between flavonoid biosynthesis and peroxidases in plant resistance to abiotic stress. Therefore, the regulatory relationship in wheat against abiotic stresses between different metabolic pathways and peroxidases still needs further study in future.

When plants experience drought for an extended period, a large number of ROS are induced. ROS exist in many forms, such as ${\text{O}}_{2}^{-}$ , H_2_O_2,_ and OH^−^ in plants (McCord, 2000; Joshi et al., 2016). The class III PRXs catalyse H_2_O_2_ reduction in the peroxidative cycle through the transfer of electrons from different donors (Hiraga et al., 2001; Gill and Tuteja, 2010). Some studies have revealed that class III PRXs play an important role in enhancing tolerance to stresses through the regulation of ROS balance in plants. For example, the *Oryza sativa* class III PRX gene *OsPRX38* activates the antioxidant system and scavenges H_2_O_2_ to enhance arsenic (As) tolerance (Maria et al., 2019). Furthermore, plants have evolved a complex antioxidant system (involving SOD, CAT, and PRX) to protect cells from damage by maintaining the balance of ROS levels (Dat et al., 2000; Gill and Tuteja, 2010). Our results showed that *TaPRX-2A* enhanced transgenic plant tolerance to drought stress by increasing antioxidant enzyme activity, thereby reducing ${\text{O}}_{2}^{-}$ and H_2_O_2_ levels. Moreover, *TaPRX-2A* also increased the expression of antioxidant-related genes, such as *CuSOD*, *FeSOD*, *CAT*, and *APX*. Therefore, these results showed that *TaPRX-2A* enhanced drought tolerance by regulating the expression of antioxidant genes that affect enzyme activity. In rice, the class III PRX gene *OsPrx30* mediates rice bacterial blight (*Xanthomonas oryzae* pv*. oryzae*)-induced ROS accumulation by the AT-hook protein *OsATH1* (Liu et al., 2021). Do wheat class III PRXs also respond to environmental stresses in this way? Future research will explore the mechanisms through which transcription factors regulate *TaPRX-2A* and regulate other antioxidant-encoding genes.

The signalling pathway of the plant hormone ABA is central to abiotic stress responses, and ABA is involved in a variety of cross-regulatory networks in response to stresses. For example, the transcription of the *Arabidopsis* dehydration-responsive gene *RD22* may be induced by ABA (Shinozaki et al., 2003). In addition, it was reported that ABA can induce the expression of dehydrin (DE)- and thaumatin-like protein (*TLP*)-encoding genes, which are essential for tolerance to abiotic stresses (Jung et al., 2005; Seo and Park, 2016). It has also been reported that ABA is involved in the PRX-mediated stress response. For example, the ABA signalling pathway controls the expression of class III PRX genes in *Tamarix hispida* (Gao et al., 2012). We previously identified that *TaPRX-2A* enhanced wheat tolerance to salt stress through activation of the ABA signalling pathway and expression of stress-related genes. Similar to the function of *TaPRX-2A* in response to salt stress, *TaPRX-2A* also improved transgenic wheat drought tolerance by altering the expression of genes involved in the ABA signalling pathway and stress-related genes, including *RD22*, *TLP4*, *GLP4*, and *GST22*, in our work. Further study is needed to explore the cross-talk regulatory mechanisms through which *TaPRX-2A* regulates the ABA signalling pathway and stress-related genes under various stresses.

Finally, we also preliminarily explore the SNP distributions of *TaPRX-2A* in different wheat cultivars and found 23 SNPs (16 in the promoter, 7 in the coding DNA sequence (CDS), and 3 in the 5’-untranslated region (UTR)) in the *TaPRX-2A* sequences. Among the 7 SNPs in the CDS, we diagrammed the four SNPs that cause changes in amino acids. The 16 SNPs in promoter influenced only a few *cis*-acting elements. Therefore, it will be interesting to explore the question about what kind of variations caused the different expression patterns of *TaPRX-2A* in different cultivars merits further consideration in subsequent investigations. In the future, *TaPRX-2A* can be used to develop SNP markers or GWAS for screening drought-resistant wheat varieties. This work will have strong application value for the cultivation of the drought-tolerant wheat varieties in the future, which is especially relevant given the anticipated hydrodynamics changes associated with ongoing climate change.

## Conclusion

In this study, we evaluated drought tolerance of various wheat cultivars and identified a class III PRXs gene *TaPRX-2A* in response to drought stress by RNA-Seq analysis in wheat. The overexpression of *TaPRX-2A* enhanced transgenic wheat tolerance against drought stress through improving antioxidant enzymes activity, activating ABA signaling pathway, and regulating stress-related genes expression, resulting in lower ROS accumulation. This work and its findings have strong future application value in the cultivation of drought-tolerant wheat varieties, which is especially relevant given the anticipated crop losses associated with the future impacts of climate change.

## Data Availability

The datasets presented in this study can be found in online repositories. The names of the repository/repositories and accession number(s) can be found below: https://www.ncbi.nlm.nih.gov/search/all/?term=PRJNA914511. The accession number of the raw data is PRJNA914511.

## Supplementary material

Supplementary Figure 1 The comparison of drought tolerance in 15 different wheat cultivars. **(A)** The phenotype of drought tolerance in different wheat cultivars was observed at 21 days after drought stress treatments. **(B)** The survive rate. Each treatment included at least 15 plants.

Supplementary Figure 2 Overview of transcriptome changes in wheat treated with drought stress. **(A)** Hierarchical clustering of DEGs in drought‐treated and control wheat. FPKM expression values were used to create the heatmap. **(B)** Number of DEGs of Liangxing 66-CK vs Liangxing 66-D, Liangxing 66-CK vs Ziyou 5-CK, Ziyou 5-CK vs Ziyou 5-D and Liangxing 66-D vs Ziyou 5-D between control and drought treatment, respectively. **(C)** Venn diagram among Liangxing 66-CK vs Liangxing 66-D, Liangxing 66-CK vs Ziyou 5-CK, Ziyou 5-CK vs Ziyou 5-D and Liangxing 66-D vs Ziyou 5-D. **(D)** Principal component analysis (PCA) of transcriptomedata obtained from drought‐treated and control wheat. Each sample included three biological replicates.

Supplementary Figure 3 Transcriptomic analysis reveals the effect of drought stress in wheat. **(A)** GO classification diagram of DEGs of Liangxing 66-CK vs Liangxing 66-D in biological process (BP), cellular component (CC) and molecular function (MF). **(B)** GO classification diagram of DEGs of Ziyou 5-CK vs Ziyou 5-D in BP, CC and MF. **(C)** KEGG enrichment dotplot of DEGs of Liangxing 66-CK vs Liangxing 66-D. **(D)** KEGG enrichment dotplot of DEGs of Ziyou 5-CK vs Ziyou 5-D.

Supplementary Figure 4 The relative expression of flavonoid biosynthesis genes. *18SrRNA* was as an endogenous control. The gene relative expression was calculated by the cycle threshold (Ct) values using formula 2^–ΔΔCT^. All experiments included three replicates and the data present the mean ± SD.

Supplementary Figure 5 Expression levels of ABA biosynthesis and signal genes. *18SrRNA* was as an endogenous control. The gene relative expression was calculated by the cycle threshold (Ct) values using formula 2^–ΔΔCT^. All experiments included three replicates and the data present the mean ± SD.

Supplementary Figure 6 Effects of drought stress on SA, JA, and IAA biosynthesis and signal transduction in wheat. Overview of SA, JA, and IAA biosynthesis and signal transduction. The green and red represent doen-regulated and up-regulated gene expression under drought stress. *AROK*, shikimate kinase; *AROA*, 3-phosphoshikimate 1-carboxyvinyltransferase; *AROC*, chorismate synthase; *TPR3*, anthranilate synthase; *TRPA*, tryptophan synthase alpha chain; *TRPB*, tryptophan synthase beta chain; *DDC*, aromatic-L-amino-acid; *AAO*, indole-3-acetaldehyde oxidase; *YUCCA*, indole-3-pyruvate monooxygenase; *trpD*, anthranilate phosphoribosyltransferase; *TRP1*, anthranilate synthase; *DDC*, L-tryptophan decarboxylase; *TAA1*, L-tryptophan—pyruvate aminotransferase; *ISS1*, aromatic aminotransferase; *ALDH*, aldehyde dehydrogenase (NAD+); *AANAT*, arylalkylamine N-acetyltransferase; *ASMT*, acetylserotonin O-methyltransferase, *AMI*, amidase; *PLA2G16*, HRAS-like suppressor 3; *ISS1*, aromatic aminotransferase; *LOX*, lipoxygenase; *AOS*, hydroperoxide dehydratase; *AOC*, allene oxide cyclase; *OPR*, 12-oxophytodienoic acid reductase; *PLA2G*, secretory phospholipase A2; *HPL*, hydroperoxide lyase; *DOX*, fatty acid alpha-dioxygenase; *OPCL1*, OPC-8:0 CoA ligase 1; *ACOX*, acyl-CoA oxidaseL; *MFP2*, enoyl-CoA hydratase/3-hydroxyacyl-CoA dehydrogenase; *FAD*, acetyl-CoA acyltransferase; *JAM*, jasmonate O-methyltransferase; *JAZ*, jasmonate ZIM domain-containing protein; *aroC*, chorismate synthase; *aroA*, 3-phosphoshikimate 1-carboxyvinyltransferase; *AUX1*, auxin influx carrier; *TIR1*, transport inhibitor response 1; *AUX/IAA*, auxin-responsive protein; *ARF*, auxin response factor; *GH3*, auxin responsive GH3 gene family; *SAUR*, SAUR family protein; *PR1*, pathogenesis-related protein 1.

Supplementary Figure 7 The relative expression of genes involved in phytohormones (SA, IAA, and JA) pathways. *18SrRNA* was as an endogenous control. The gene relative expression was calculated by the cycle threshold (Ct) values using formula 2^–ΔΔCT^. All experiments included three replicates and the data present the mean ± SD.

Supplementary Figure 8 The relative expression of genes involved in phenolamides and antioxidant pathway. *18SrRNA* was as an endogenous control. The gene relative expression was calculated by the cycle threshold (Ct) values using formula 2^–ΔΔCT^. All experiments included three replicates and the data present the mean ± SD.

Supplementary Figure 9 The expression profile of *TaPRX-2A* among wheat cultivars with different levels of drought tolerance. *18SrRNA* was as an endogenous control. The gene relative expression was calculated by the cycle threshold (Ct) values using formula 2^–ΔΔCT^. All experiments included three replicates and the data present the mean ± SD.

Supplementary Figure 10 The expression profile of stress-related genes in *TaPRX-2A*-overexpressing transgenic lines and WT plants under drought stress. (A) The expression levels of stress-related genes in transgenic lines and WT plants under drought stress. (B) The expression levels of ABA-related genes in transgenic lines and WT plants. *18SrRNA* was as an endogenous control. The gene relative expression was calculated by the cycle threshold (Ct) values using formula 2^–ΔΔCT^. All experiments included three replicates and the data present the mean ± SD.

Supplementary Table 1 EXECL file with RNA-Seq data.

Supplementary Table 2 DEGs involved in flavonoid biosynthesis pathway, phytohormones, and phenolamides and antioxidant biosynthesis pathways.

Supplementary Table 3 Primers used for analysis.
