# Corrigendum: Comparative transcriptomic analysis and functional characterization reveals that the class III peroxidase gene *TaPRX-2A* regulates drought stress tolerance in transgenic wheat

**DOI:** 10.3389/fpls.2025.1565141

**Published:** 2025-03-13

**Authors:** Peisen Su, Chao Sui, Yufei Niu, Jingyu Li, Shuhan Wang, Fanting Sun, Jun Yan, Shangjing Guo

**Affiliations:** ^1^ College of Agronomy, Liaocheng University, Liaocheng, China; ^2^ Key Laboratory of Huang-Huai-Hai Smart Agricultural Technology of the Ministry of Agriculture and Rural Affairs, College of Information Science and Engineering, Shandong Agricultural University, Tai’an, Shandong, China

**Keywords:** transcriptomics, drought tolerance, class III peroxidase, TaPRX-2A, ROS

In the published article, there was an error in **Figure 6A**, **Figure 7C** as published. In **Figure 6A**, the representative photo of TaOE2 was duplicated and used as TaOE3 at 0 day. In **Figure 7C**, the representative photo of TaOE2 was duplicated and used as TaOE3. The corrected **Figure 6A** and its caption “**Figure 6** *TaPRX-2A* overexpression increased the drought tolerance. (A) Phenotype of *TaPRX-2A*-overexpressing transgenic and WT wheat (the cultivar “KN199”) with drought treatment. (B) Survival rates of *TaPRX-2A*-overexpressing transgenic lines and WT wheat. (C) shoot length of *TaPRX-2A*-overexpressing transgenic lines and WT wheat. (D) Relative water content (RWC), and (E) root length. (F) MDA content of *TaPRX-2A*-overexpressing transgenic lines and WT wheat. (G) soluble sugar content of *TaPRX-2A*-overexpressing transgenic lines and WT wheat. (H) proline content, and (I) soluble protein content of *TaPRX-2A*-overexpressing and WT plants. All experiments included three replicates and the data present the mean ± SD. *P < 0.05 and **P < 0.01 indicate a significant difference compared with WT.” and the corrected **Figure 7C** along with its caption “**Figure 7** Analysis of ROS scavenging capacity andantioxidant enzymes activity in transgenic wheat lines. (A) Detection of O_2_^−^ generation by NBT staining and O_2_^−^ content (B). (C) Detection of H_2_O_2_ accumulation by DAB staining and H_2_O_2_ content (D). (E) Detection of SOD activity in *TaPRX-2A*-overexpressing transgenic lines and WT wheat. (F) Detection of CAT activity in *TaPRX-2A*-overexpressing transgenic lines and WT wheat. (G) Detection of POD activity in *TaPRX-2A*-overexpressing transgenic lines and WT wheat. All experiments included three replicates and the data present the mean ± SD. *P < 0.05 and **P < 0.01 indicate a significant difference compared with WT.” appear below.

**Figure 6:**
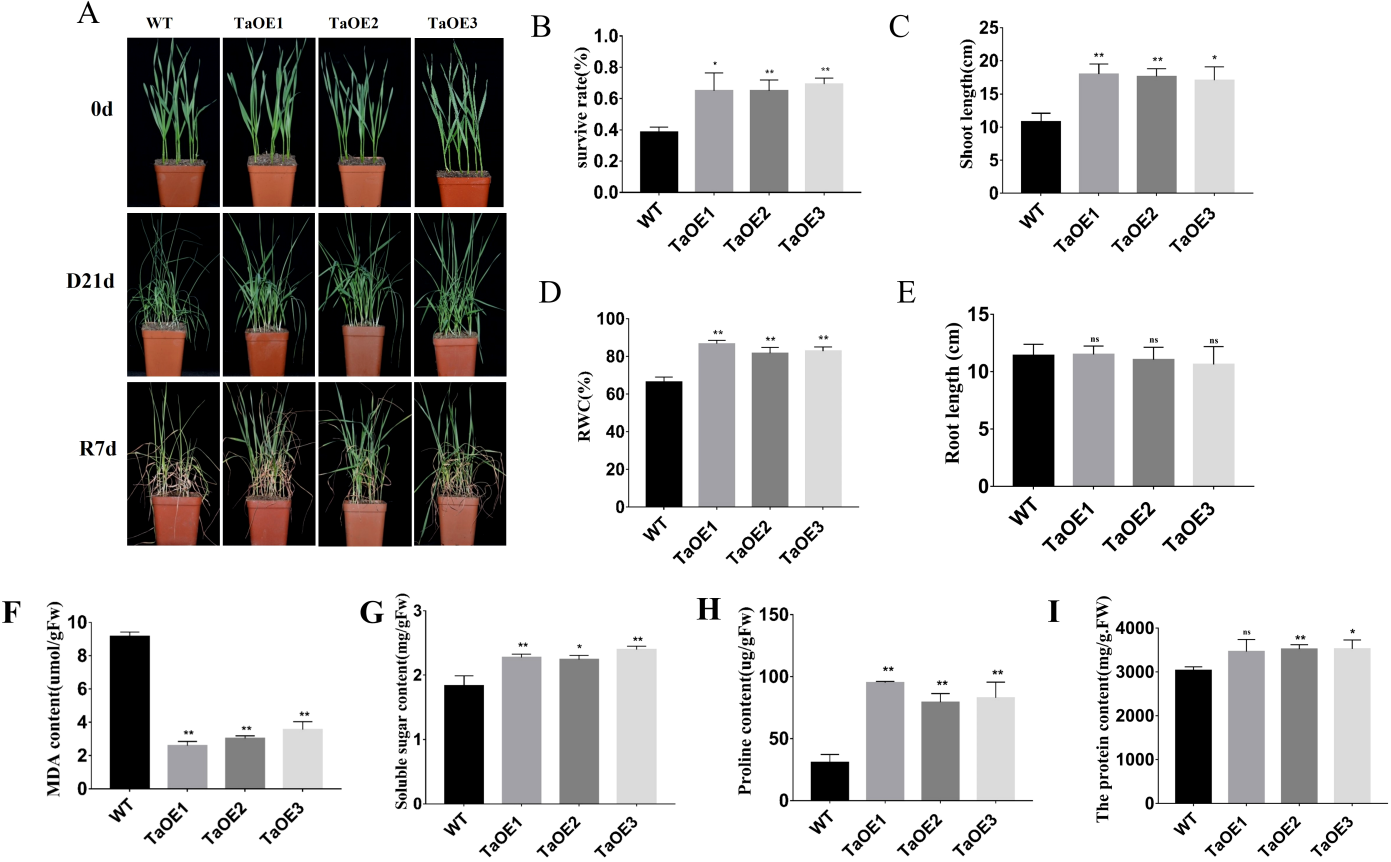
*TaPRX-2A* overexpression increased the drought tolerance. **(A)** Phenotype of *TaPRX-2A*-overexpressing transgenic and WT wheat (the cultivar “KN199”) with drought treatment. **(B)** Survival rates of *TaPRX-2A*-overexpressing transgenic lines and WT wheat. **(C)** shoot length of *TaPRX-2A*-overexpressing transgenic lines and WT wheat. **(D)** Relative water content (RWC), and **(E)** root length. **(F)** MDA content of *TaPRX-2A*-overexpressing transgenic lines and WT wheat. **(G)** soluble sugar content of *TaPRX-2A*-overexpressing transgenic lines and WT wheat. **(H)** proline content, and **(I)** soluble protein content of *TaPRX-2A*-overexpressing and WT plants. All experiments included three replicates and the data present the mean ± SD. *P < 0.05 and **P < 0.01 indicate a significant difference compared with WT. The “ns” presents “no differences”.

**Figure 7:**
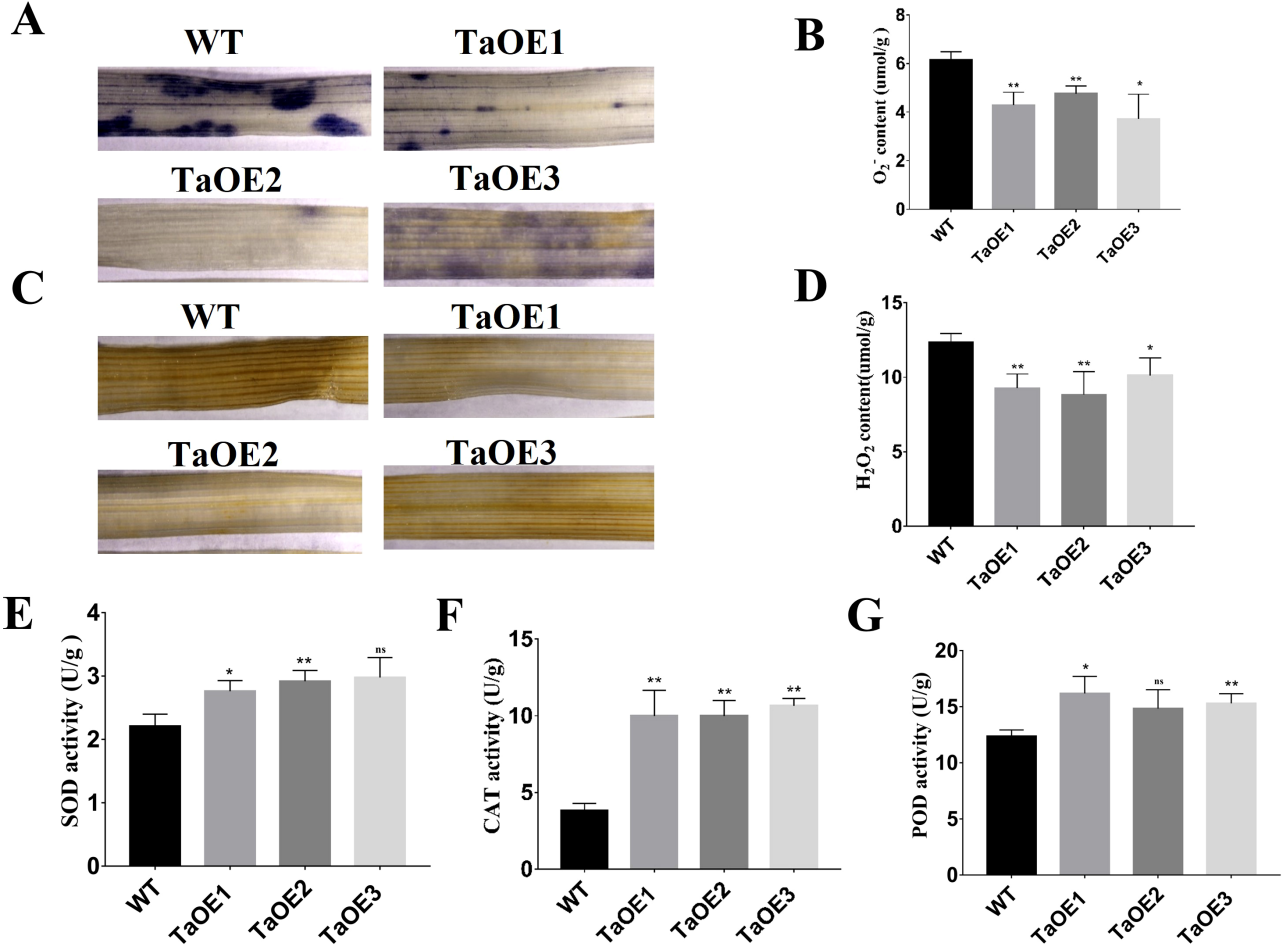
Analysis of ROS scavenging capacity andantioxidant enzymes activity in transgenic wheat lines. **(A)** Detection of ${\text{O}}_{2}^{–}$ generation by NBT staining and ${\text{O}}_{2}^{–}$ content **(B)**. **(C)** Detection of H_2_O_2_ accumulation by DAB staining and H_2_O_2_ content **(D)**. **(E)** Detection of SOD activity in *TaPRX-2A*-overexpressing transgenic lines and WT wheat. **(F)** Detection of CAT activity in *TaPRX-2A*-overexpressing transgenic lines and WT wheat. **(G)** Detection of POD activity in *TaPRX-2A*-overexpressing transgenic lines and WT wheat. All experiments included three replicates and the data present the mean ± SD. *P < 0.05 and **P < 0.01 indicate a significant difference compared with WT. The “ns” presents “no differences”.

The authors apologize for this error and state that this does not change the scientific conclusions of the article in any way. The original article has been updated.

