# Supplementary figures and images for: Comparative transcriptomic analysis and functional characterization reveals that the class III peroxidase gene TaPRX-2A regulates drought stress tolerance in transgenic wheat
Source: Front Plant Sci. 2023 Feb 15;14:1119162. doi: 10.3389/fpls.2023.1119162 (PMC9976582; doi:10.3389/fpls.2023.1119162)

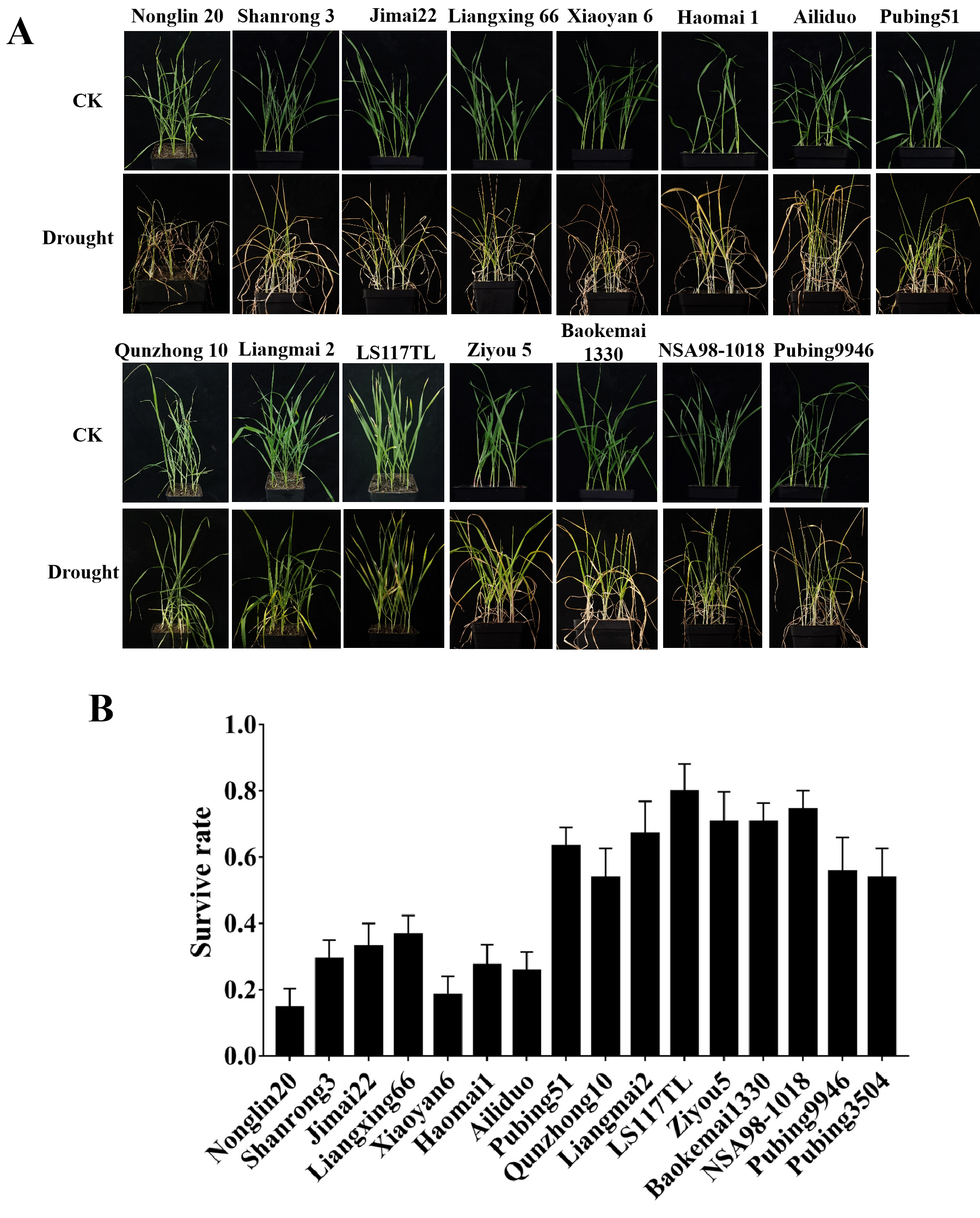

Supplement: Supplementary Figure 1 — The comparison of drought tolerance in 15 different wheat cultivars. (A) The phenotype of drought tolerance in different wheat cultivars was observed at 21 days after drought stress treatments. (B) The survive rate. Each treatment included at least 15 plants. [file Image1.jpeg]

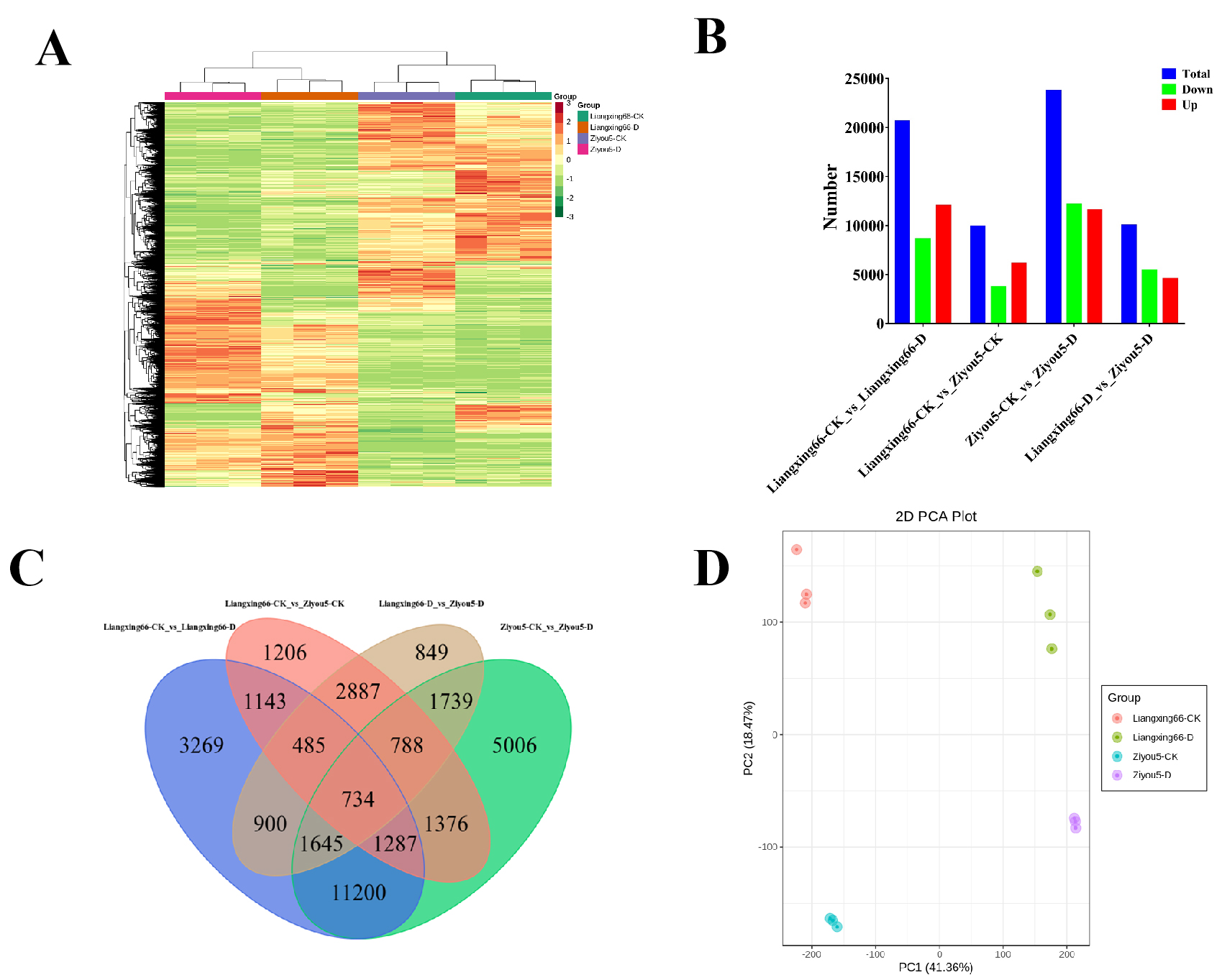

Supplement: Supplementary Figure 2 — Overview of transcriptome changes in wheat treated with drought stress. (A) Hierarchical clustering of DEGs in drought‐treated and control wheat. FPKM expression values were used to create the heatmap. (B) Number of DEGs of Liangxing 66-CK vs Liangxing 66-D, Liangxing 66-CK vs Ziyou 5-CK, Ziyou 5-CK vs Ziyou 5-D and Liangxing 66-D vs Ziyou 5-D between control and drought treatment, respectively. (C) Venn diagram among Liangxing 66-CK vs Liangxing 66-D, Liangxing 66-CK vs Ziyou 5-CK, Ziyou 5-CK vs Ziyou 5-D and Liangxing 66-D vs Ziyou 5-D. (D) Principal component analysis (PCA) of transcriptomedata obtained from drought‐treated and control wheat. Each sample included three biological replicates. [file Image2.jpeg]

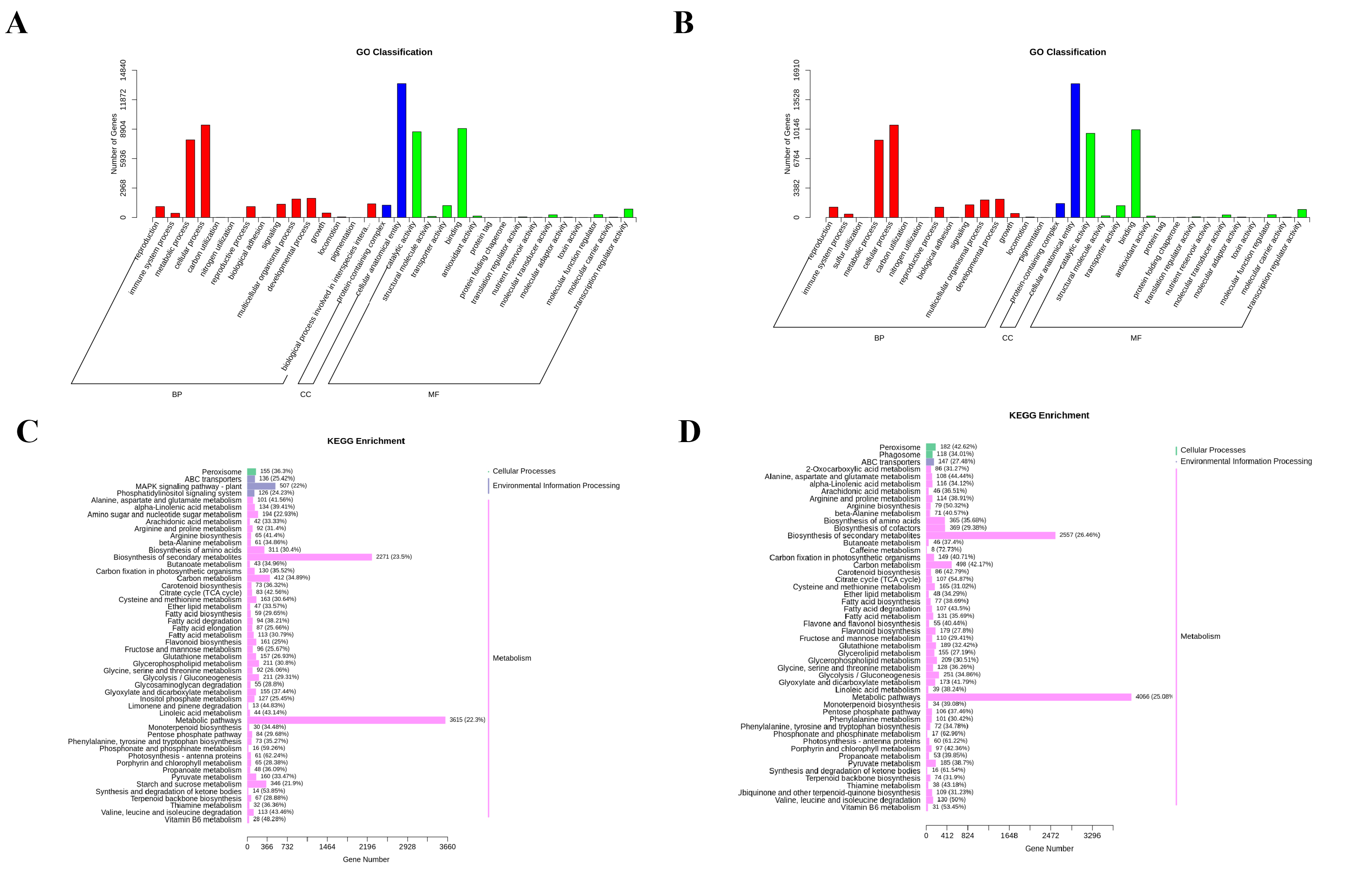

Supplement: Supplementary Figure 3 — Transcriptomic analysis reveals the effect of drought stress in wheat. (A) GO classification diagram of DEGs of Liangxing 66-CK vs Liangxing 66-D in biological process (BP), cellular component (CC) and molecular function (MF). (B) GO classification diagram of DEGs of Ziyou 5-CK vs Ziyou 5-D in BP, CC and MF. (C) KEGG enrichment dotplot of DEGs of Liangxing 66-CK vs Liangxing 66-D. (D) KEGG enrichment dotplot of DEGs of Ziyou 5-CK vs Ziyou 5-D. [file Image3.jpeg]

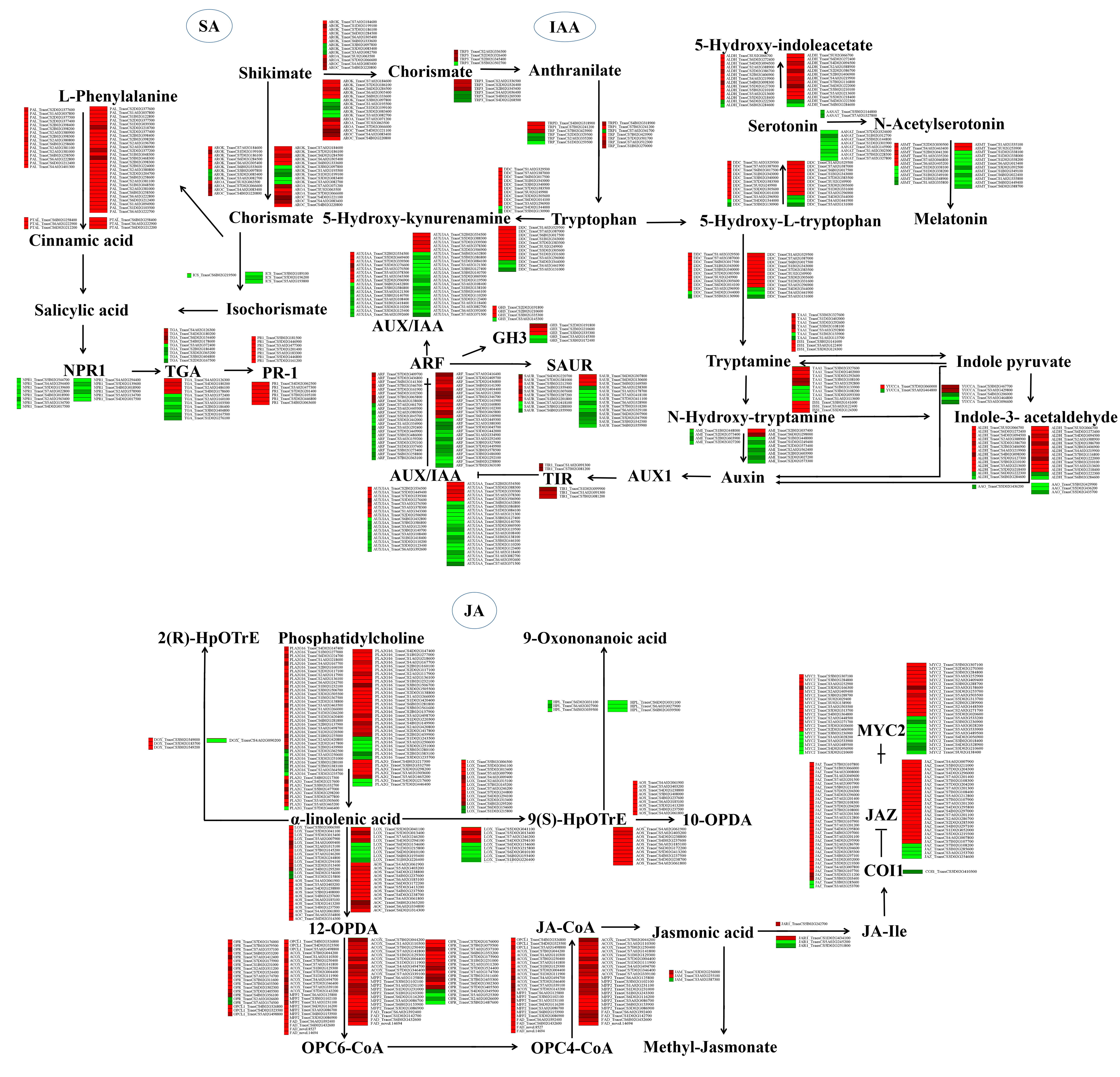

Supplement: Supplementary Figure 6 — Effects of drought stress on SA, JA, and IAA biosynthesis and signal transduction in wheat. Overview of SA, JA, and IAA biosynthesis and signal transduction. The green and red represent doen-regulated and up-regulated gene expression under drought stress. AROK, shikimate kinase; AROA, 3-phosphoshikimate 1-carboxyvinyltransferase; AROC, chorismate synthase; TPR3, anthranilate synthase; TRPA, tryptophan synthase alpha chain; TRPB, tryptophan synthase beta chain; DDC, aromatic-L-amino-acid; AAO, indole-3-acetaldehyde oxidase; YUCCA, indole-3-pyruvate monooxygenase; trpD, anthranilate phosphoribosyltransferase; TRP1, anthranilate synthase; DDC, L-tryptophan decarboxylase; TAA1, L-tryptophan—pyruvate aminotransferase; ISS1, aromatic aminotransferase; ALDH, aldehyde dehydrogenase (NAD+); AANAT, arylalkylamine N-acetyltransferase; ASMT, acetylserotonin O-methyltransferase, AMI, amidase; PLA2G16, HRAS-like suppressor 3; ISS1, aromatic aminotransferase; LOX, lipoxygenase; AOS, hydroperoxide dehydratase; AOC, allene oxide cyclase; OPR, 12-oxophytodienoic acid reductase; PLA2G, secretory phospholipase A2; HPL, hydroperoxide lyase; DOX, fatty acid alpha-dioxygenase; OPCL1, OPC-8:0 CoA ligase 1; ACOX, acyl-CoA oxidaseL; MFP2, enoyl-CoA hydratase/3-hydroxyacyl-CoA dehydrogenase; FAD, acetyl-CoA acyltransferase; JAM, jasmonate O-methyltransferase; JAZ, jasmonate ZIM domain-containing protein; aroC, chorismate synthase; aroA, 3-phosphoshikimate 1-carboxyvinyltransferase; AUX1, auxin influx carrier; TIR1, transport inhibitor response 1; AUX/IAA, auxin-responsive protein; ARF, auxin response factor; GH3, auxin responsive GH3 gene family; SAUR, SAUR family protein; PR1, pathogenesis-related protein 1. [file Image6.jpeg]

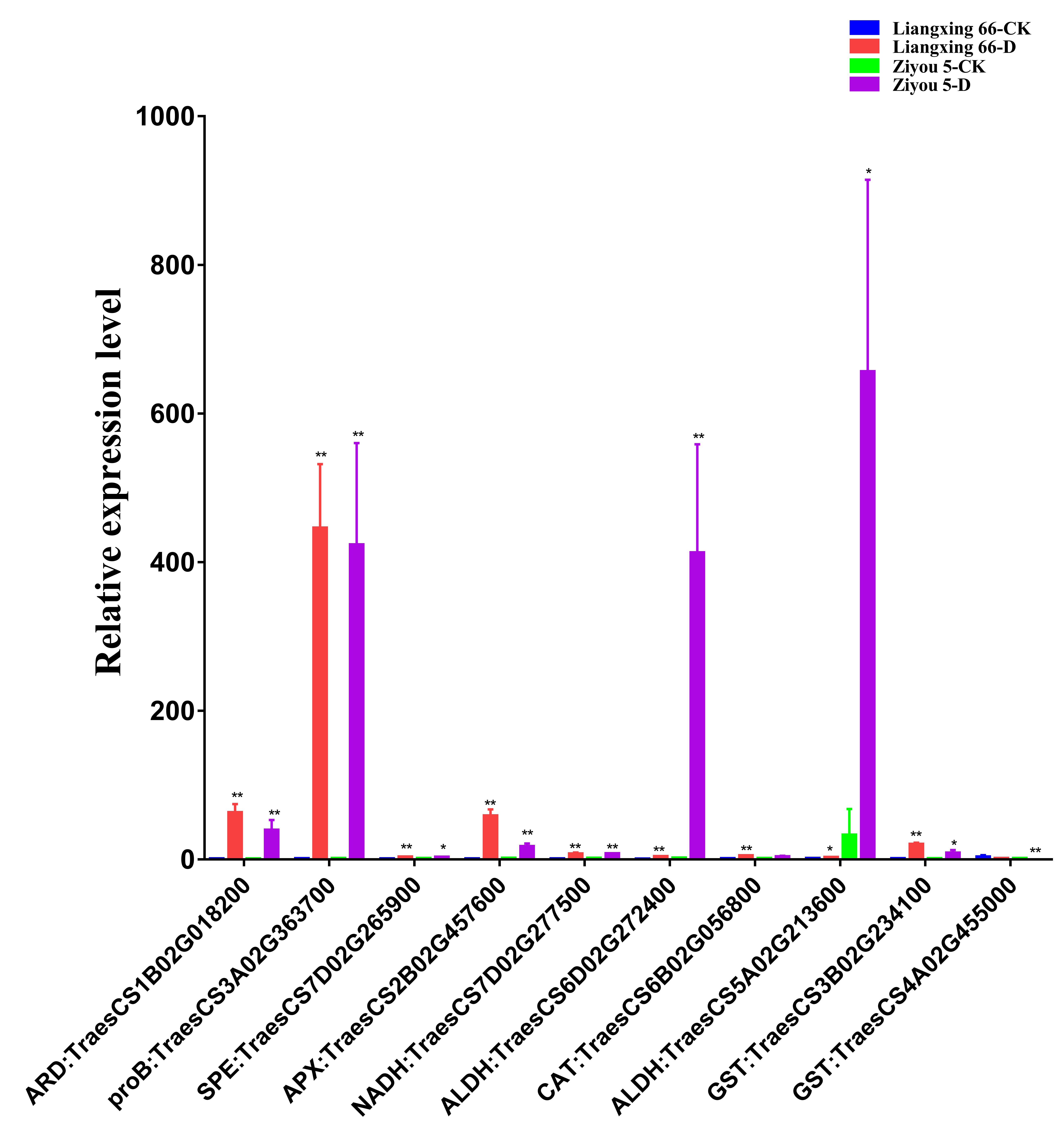

Supplement: Supplementary Figure 8 — The relative expression of genes involved in phenolamides and antioxidant pathway. 18SrRNA was as an endogenous control. The gene relative expression was calculated by the cycle threshold (Ct) values using formula 2–ΔΔCT. All experiments included three replicates and the data present the mean ± SD. [file Image8.jpeg]

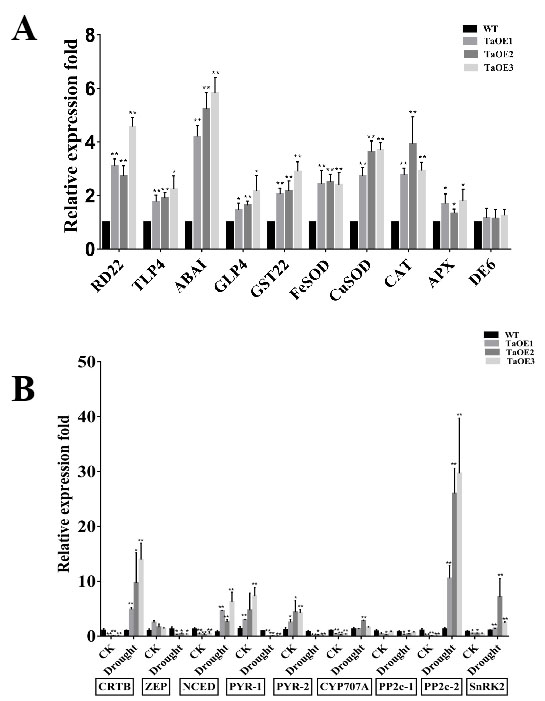

Supplement: Supplementary Figure 10 — The expression profile of stress-related genes in TaPRX-2A-overexpressing transgenic lines and WT plants under drought stress. (A) The expression levels of stress-related genes in transgenic lines and WT plants under drought stress. (B) The expression levels of ABA-related genes in transgenic lines and WT plants. 18SrRNA was as an endogenous control. The gene relative expression was calculated by the cycle threshold (Ct) values using formula 2–ΔΔCT. All experiments included three replicates and the data present the mean ± SD. [file Image10.jpeg]
